# Dietary Impacts on Gestational Diabetes: Connection between Gut Microbiome and Epigenetic Mechanisms

**DOI:** 10.3390/nu14245269

**Published:** 2022-12-10

**Authors:** Taiwo Bankole, Hung Winn, Yuanyuan Li

**Affiliations:** 1 Department of Nutrition and Food Science, University of Maryland, College Park, MD 20742, USA; 2 Department of Obstetrics, Gynecology and Women’s Health, University of Missouri, Columbia, MO 65212, USA

**Keywords:** maternal diets, gestational diabetes, microbiome, metabolome, epigenome

## Abstract

Gestational diabetes mellitus (GDM) is one of the most common obstetric complications due to an increased level of glucose intolerance during pregnancy. The prevalence of GDM increases due to the obesity epidemic. GDM is also associated with an increased risk of gestational hypertension and preeclampsia resulting in elevated maternal and perinatal morbidity and mortality. Diet is one of the most important environmental factors associated with etiology of GDM. Studies have shown that the consumption of certain bioactive diets and nutrients before and during pregnancy might have preventive effects against GDM leading to a healthy pregnancy outcome as well as beneficial metabolic outcomes later in the offspring’s life. Gut microbiome as a biological ecosystem bridges the gap between human health and diseases through diets. Maternal diets affect maternal and fetal gut microbiome and metabolomics profiles, which consequently regulate the host epigenome, thus contributing to later-life metabolic health in both mother and offspring. This review discusses the current knowledge regarding how epigenetic mechanisms mediate the interaction between maternal bioactive diets, the gut microbiome and the metabolome leading to improved metabolic health in both mother and offspring.

## 1. Introduction

Gestational diabetes mellitus (GDM) is a common obstetric metabolic complication where women without diabetes before pregnancy show increased glucose levels and insulin resistance during pregnancy. According to the International Diabetes Federation (IDF), GDM has a global prevalence, with the highest prevalence in the Middle East and North Africa [1,2]. Globally, maternal GDM affects about one in six pregnancies. Extensive studies have reported a high rate of hyperglycemia and hyperinsulinemia among GDM women [3,4].

It is well established that the intrauterine environment impacts offspring’s health throughout their lifespan [5]. During development, fetal growth is greatly influenced by the placental function, which serves as a biological link between the mother and the developing fetus through nutrient supply [6]. Periconceptional and prenatal exposure to certain environmental factors that disrupt placental function might lead to detrimental health outcomes [7]. Importantly, maternal nutritional factors have been reported to influence offspring disease susceptibility [8,9]. Likewise, maternal nutrition and dietary status crucially affects GDM incidence by regulating maternal, fetal, and neonatal glycemic and insulin statuses during and after pregnancy [10].

Fetal development in utero is influenced by epigenetic regulations, such as DNA methylation, histone modifications, and non-coding RNAs. These mechanisms are involved in the activation or repression of developmental genes required for the epigenomic reprogramming process during early development [11]. Most of these epigenetic landmarks are dynamically changed in response to environmental factors, such as diets, that may influence the offspring’s health outcomes later in life [8]. Maternal exposure to certain diets that contain bioactive compounds with epigenetic regulatory effects (so called “epigenetics diets”) can regulate epigenetic mechanisms and influence fetal health outcomes, including metabolic syndrome [9,12,13]. Intriguingly, our recent studies showed that bioactive dietary components, such as genistein in soy and sulforaphane in broccoli sprouts, can significantly reduce the risk of breast cancer and metabolic disorders in the offspring by modulating differential gene expression during early embryonic development [14,15,16]. This transgenerational effect can be mediated through epigenetics mechanisms via the maternal–fetal link of the intrauterine environment bridged by maternal diets. Therefore, early-life epigenetic changes through maternal diets may affect the offspring’s disease susceptibility and metabolism later in life [17].

Maternal dietary factors play a role in metabolic disorders through intestinal microbes [18,19]. Women with GDM have altered microbial composition compared to lean women without GDM [20,21]. Gut microbial imbalance is a significant feature for GDM, type 1 and 2 diabetes, as well as obesity [19]. Healthy and balanced fetal gut microbiota has a long-lasting impact on offspring by mitigating the risks of metabolic disorders later in life. Maternal nutritional factors can regulate early-life the microbial colonization, succession, and function of fetal and neonatal microbiota composition, which can significantly affect offspring health conditions later in life [21].

An alternative pathway that regulates fetal health, growth, and development aside from maternal gut microbiota is the transport of microbial-derived metabolites through the uteroplacental unit [18]. For instance, beneficial microbial metabolites, short chain fatty acids, are responsible for maintaining normal glucose levels and insulin signaling during pregnancy [20]. Notably, maternal nutritional factors that can influence maternal–fetal vertical transmission of gut microbiota and metabolites can regulate fetal and neonatal metabolism [22,23,24,25]. Thus, maternal nutritional intervention via the regulation of intestinal microbiome and metabolomics can create an effective therapeutic avenue for metabolic disorders for both GDM mothers and their affected progenies [26].

Interestingly, research on the precise mechanisms through which microbiome-metabolome interface induces epigenetic reprogramming via maternal diets has exponentially increased in recent years [20]. Maternal intake of bioactive dietary components with an epigenetic modulatory property is believed to have a beneficial impact on fetal epigenome establishment, subsequent gene expression profiles, and early development in utero [11,27]. Therefore, appropriate maternal nutrition exposure may lead to a reduced risk of GDM and metabolic diseases in their newborns [28,29]. Thus, enhanced understanding of the interrelationships between maternal dietary composition, intestinal microbes, metabolites, and epigenetics would provide a valuable translational insight into GDM and its obstetric sequelae.

## 2. Gestational Diabetes Mellitus (GDM)

GDM develops during pregnancy and usually resolves after birth. It is normally due to glucose intolerance and beta cell dysfunction that adversely affect the health of both mothers and their offspring [30]. GDM affects about 9–25% of pregnancies globally. Importantly, it is used as a risk factor of type 2 diabetes, obesity, and other metabolic comorbidities among pregnant women [5]. Screening for GDM is usually done between 24 weeks and 28 weeks of gestation using a 50 g/1 h oral glucose challenge test (GCT) and a subsequent confirmatory test of 100 g/3 h glucose tolerance test (GTT) if the GCT plasma glucose value is ≥7.7 mmol/L (130–140 mg/dL) [3,4]. GDM is also distinctively connected to maternal inflammation and placental malfunction [31,32]. Some altered molecular pathways in GDM include nuclear factor-κB (NF-κB), peroxisome proliferator-activated receptors (PPARs), sirtuins (SIRTs), PI3 K/mTOR, glycogen synthase kinase 3 (GSK3), adenosine monophosphate (AMP)-activated protein kinase (AMPK), inflammasome, and endoplasmic reticulum (ER) stress [32]. Potential biomarkers of GDM, such as adiponectin, TNF-alpha (TNF-α), leptin, interleukin-6, resistin, visfatin, and apelin, have been associated with lipid metabolism and insulin sensitivity dynamics. For instance, TNF-α increases insulin resistance by affecting the insulin receptor and subsequent insulin signaling cascades [14].

Although these obstetric complications most likely resolve after pregnancy [8], about 50% of GDM women have an increased risk of developing type 2 diabetes later in life, especially when there is increased postpartum weight gain [14,33]. Obesity is a very high risk for GDM due to glucose insensitivity and insufficient insulin response [34,35,36]. GDM not only impairs glucose metabolism but also affects lipid metabolism. Increased triglyceride and cholesterol concentrations as gestation progresses are more pronounced in GDM compared to normoglycemic pregnant women [37]. Other risk factors for GDM include advanced maternal age, gravidity, parity, ethnicity and racial groups, genetics polymorphism, environmental influences, and socio-economic status [5].

### 2.1. The Influence of GDM on Maternal Health and Pregnancy Outcomes

**Figure 1:**
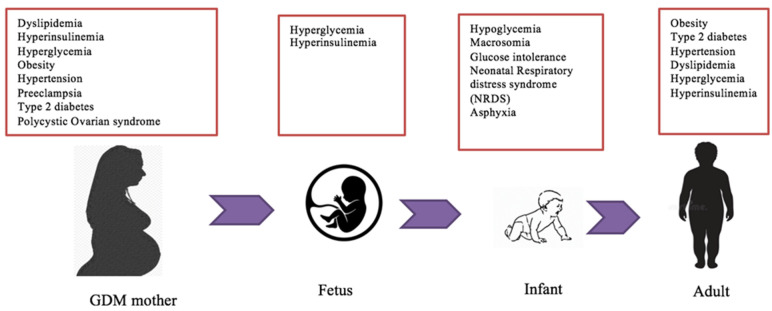
Metabolic complications associated with GDM. Several metabolic risks link maternal GDM to fetal and neonatal health outcomes in utero. These obstetrical comorbidities influence fetal growth and development from embryogenesis to offspring adulthood and are mostly caused by dysfunction in glucose, insulin, and lipid pathways. The majority of these clinical conditions in GDM mothers are developed and persistent in the offspring later in life.

GDM may result in a transient or long-term impact on maternal health and pregnancy outcomes (Figure 1). Maternal weight gain and increased BMI during pregnancy are associated with GDM susceptibility [3,38]. However, a small proportion of GDM mothers have normal body weight and BMI. This observation suggests that other factors, such as unhealthy diets and sedentary lifestyles pre- and during pregnancy, may also impose the risk of GDM [38]. Obesity and GDM are strongly associated with an increased risk of fetal macrosomia. Fetal macrosomia is defined as estimated fetal weight being at least 4000 g or greater than the 90th percentile for gestational age [39]. It affects 12% of newborns with mothers without GDM and 15–45% of GDM mothers [40,41]. In addition, women with pre-pregnancy obesity, excessive gestational weight gain (GWG), and GDM are susceptible to an increased risk of caesarean section delivery [42]. Other maternal complications associated with fetal macrosomia include vaginal lacerations, perineal tears, postpartum hemorrhage, prolonged labor, uterine rupture, infection, and maternal mortality [36,40,43].

Furthermore, the role of the placenta in GDM women is equally important for fetal development [33]. Notably, as placenta size increases, placenta associated hormones, such as estrogen, progesterone, lactogen, and cortisol increase in maternal circulation [40,44]. Lactogen decreases insulin sensitivity and stimulates lipolysis during gestation. Thus, increased free fatty acids (FFAs), which are a useful energy source for maternal needs in late gestation, contribute to fetal growth and increased adiposity in GDM pregnancies [36]. In addition, hyperinsulinemia is frequently observed among GDM mothers as opposed to hypo- and normo-insulinemia in non-GDM control mothers [3,36]. GDM women significantly express high levels of glucose, triglycerides, leptin, lipocalin-2, and c-peptide, but they express low adiponectin levels during their first trimester [45,46]. In addition, GDM increases the risks of preeclampsia, type 2 diabetes, hypertension, polycystic ovarian syndrome (PCOS), and breast cancer in mothers’ later life [3,36,47].

### 2.2. The influence of GDM on Fetal Development and Offspring Metabolic Outcomes

GDM is strongly associated with abnormal fetal growth and development as well as dysregulated metabolic programming that affects offspring’s health (Figure 1). Offspring from GDM mothers are highly vulnerable to experiencing childhood obesity and/or metabolic syndromes [48]. Newborns delivered by GDM obese mothers have a significant risk of hyperinsulinemia compared to those with non-obese GDM mothers [40]. Similarly, in utero exposure to maternal hyperglycemia increases the risk of obesity, glucose intolerance, and type 2 diabetes in the offspring [49,50]. Thus, the inherit tendency of obesity and type 2 diabetes mellitus from GDM mothers to offspring is a major health concern [40].

Additionally, maternal hyperglycemia due to low insulin sensitivity leads to a larger amount of glucose passing through the placenta to the fetus [40]. Thus, elevated fetal insulin secretion in response to fetal hyperglycemia results in fetal hypoglycemia which causes gluconeogenesis and fat oxidation aberrations [51]. Consequently, fetal macrosomia and accelerated fetal growth associated with maternal hyperglycemia result in obesity in the offspring [49]. Increased insulin in amniotic fluid, probably representing stimulated fetal islet development, may increase the risk of type 1 diabetes in the offspring’s early age [34]. Correspondingly, increased fat deposition in neonate’s liver from GDM mothers may increase the risk of developing non-alcoholic fatty liver disease during childhood [52]. In addition, GDM mothers’ infants have increased risks of clavicle fractures, shoulder dystocia, brachial plexus injury, respiratory problems [43], intrauterine growth restriction [50], and neonate mortality [36,53]. Metabolically, GDM mothers’ neonates have an overexpression of leptin and a decreased adiponectin production [53].

## 3. Epigenetic Reprogramming during Early Development

Epigenetic mechanisms, mainly DNA methylation, histone modification, and non-coding RNAs, have been shown to play important roles in fetal development and transgenerational inheritance [54]. Epigenetic regulation has been widely studied in gene-environmental interaction during early development [55]. Epigenetic alterations in response to maternal nutrition regulate metabolic reprogramming during embryogenesis and early development [9,13,34]. The adverse effects of maternal GDM can also be mediated through epigenetic mechanisms [56].

The placenta plays an important role in the epigenetic regulation of fetal growth and development via maternal diets [57]. Placental structure and function bridge the gap between maternal nutrition and fetal health outcomes. This can also be affected by epigenetics mechanisms, such as epigenetic imprinting and gene expression regulators [58]. A recent study by Canicais et al. reported that the increased expression of certain imprinted genes, such as DNA methyltransferases and Ten-eleven-translocation (TET) genes, can lead to fetal growth restriction and development due to inadequate placental nutrient transfer from mother to fetus [59].

### 3.1. Epigenetic Regulation in GDM

Epigenetics refers to the heritable and reversible changes to DNA and histone proteins without a variation in DNA sequences [17]. Basically, epigenetic mechanisms mediate most physiological processes, including development, genomic integrity, imprinting, gene expression, DNA replication, and nucleosome stability [60]. Disruption of epigenetic modifications are implicated in multiple pathogenesis, including cancer and metabolic disorders, such as dysregulation of lipid metabolism, insulin resistance, and inflammation, that are associated with GDM [56].

#### 3.1.1. DNA Methylation

DNA methylation is a covalent modification that involves the addition of a methyl group (CH_3_) to the fifth carbon position of cytosine base to form 5-methyl cytosine (5mC) [61]. This modification creates a gene repressive pattern by preventing transcription factors or regulatory proteins from binding to DNA [61]. The pattern of DNA methylation is catalyzed by DNA methyltransferases (DNMTs), including de novo methyltransferases (DNMT3a, DNMT3b, and non-catalytic DNMT3l) and maintenance methyltransferase (DNMT1). CpG dinucleotides are mostly found at CpG islands and are randomly distributed across the genome. Most CpG sites found at the promoters and transcription start site (TSS) are unmethylated [62]. Alternative regulators of methylation are the TET genes, which regulate demethylation by oxidizing 5mC to 5-hydroxymethylcytosine (5mhC), 5-formylcytocytosine (5fC), and 5-carboxylcytosine (5caC) utilizing base excision repair (BER) proteins [60].

Both in silico [17,63] and in vitro [64,65,66,67,68] studies have provided substantial evidence of epigenetic alterations through DNA methylation in GDM. Howe et al. analyzed the state of differentially methylated genes involved in neonatal development in maternal GDM as opposed to controlled mothers [63]. These differentially methylated regions (DMRs) (OR2L13 promoter and gene body of CYP2E1) were hypomethylated in newborns from GDM mothers. These genes were associated with autism spectrum disorders as well as type 1 and 2 diabetes, respectively. Nomura et al. evaluated the intrauterine epigenetic association of maternal GDM and placenta-regulated fetal growth and development [64]. An analysis of global methylation showed significant placental hypomethylation in GDM mothers. Likewise, GDM exposure can epigenetically influence offspring’s methylome, consequently impacting fetal metabolic programming and disease cascades [65].

Genomic imprinting is a special epigenetic process for parent-specific gene expression. Numerous imprinted genes have diverse functions in fetoplacental growth and development during embryogenesis and metabolism [67]. Imprinted genes clustered at imprinted domains are controlled by differentially methylated imprinted control regions (ICR) [68]. Petry et al. reported that a disruption of fetal imprinted H19/IGF2 and INS genes either by knockout or single nucleotide polymorphism (SNPs) were associated with higher maternal blood glucose concentration. It revealed that the influence of imprinted fetal genotype on maternal glycemic index might predispose pregnant women to GDM [69].

#### 3.1.2. Histone Modifications

Histone modification is an important epigenetic mechanism that regulates transcription and chromatin structure [70]. The eukaryotic DNA (146bp) is wrapped (1.65 turns) around the histone octamer that comprises four core histone subunits, including H2A, H2B, H3, H4, and a linker histone H1. Histone octamer and bound DNA form the nucleosome which is a functional unit of a chromatin [71]. Histone modifications primarily occur on histone protein tails at the N-terminal domain that are involved in chromatin accessibility (euchromatin) and compaction (heterochromatin) [72]. These covalent modifications exhibit several amino acids which are modified by different biochemical patterns, such as acetylation on lysine, methylation on lysine and arginine, phosphorylation on serine, and threonine and ubiquitylation. Acetylation and methylation on lysine and arginine residues of H3 and H4 are the most common histone modification patterns which play significant roles in gene activation and repression. While histone acetylation is commonly involved in chromatin activation, histone methylation can lead to either gene transcriptional activation or silencing depending on specific modified residues and methylation types. For instance, the methylation of histone H3 at lysine 4 or 36 (H3K4/K36-me3) normally regulates transcriptional activation, whereas variant specific H3K9/K27-me3 leads to gene silencing expression [73].

Several histone and chromatin modifying enzymes catalyze histone modifications. Histone acetyltransferases (HATs) regulates histone acetylation, and histone deacetylases (HDACs) catalyzes histone deacetylation process [74]. Histone lysine methyltransferases (HKMTs) and protein arginine methyltransferases (PRMTs) contain SET domains which catalyze the addition of methyl group on lysine and arginine residues, respectively. Moreover, histone demethylases catalytically remove methyl groups, such as JmjC domain-containing proteins and LSD1 [68,73]. Additionally, some HKMTs can act as epigenetically reacting subunits, such as EZH2 and MLL/ASH1, which are subunits of polycomb repressive complexes and trithorax activating complexes, respectively [75]. Hepp et al. reported a downregulation of H3K9ac expression in fetal endothelial cells, decidua, and syncytiotrophoblasts of GDM placentas compared to the controls [76]. This study indicates that histone modification may also play an important role in GDM.

#### 3.1.3. Non-Coding RNAs (ncRNAs)

Non-coding RNAs (ncRNAs) are a group of important epigenetic regulators contributing to several pathogenesis [77,78]. In eukaryotic cells, 75% of genomic DNA are transcribed, but a majority of DNA do not code for proteins. Most of these untranslated RNAs are non-coding RNA that are classified based on their functions and molecular sizes [68]. Regulatory ncRNAs include short-interfering RNAs (siRNAs), microRNAs (miRNAs), piwi-interacting RNAs (piRNAs), long non-coding RNAs (lncRNAs), and large intergenic non-coding RNAs (lincRNA) that are frequently involved in transcription, mRNA stability, and gene silencing [79]. siRNAs are double-stranded, regulatory RNA molecules (~20–24 nucleotides long) that are involved in sustaining genome integrity by silencing gene expression at specific loci. They engage HDACs and the polycomb group of proteins for heterochromatin maintenance [68]. miRNAs are small, single-stranded RNA molecules (18–24 nucleotides long) that often interact with a group of small proteins and incorporate into the RNA-induced silencing complex (RISC). Functional miRNAs in RISC down-regulate gene expression by translationally inhibiting or degrading targeted mRNA transcripts and can recruit chromatin-remodeling proteins to DNA regulatory regions for chromatin alterations [68,80]. LncRNAs (>200 nucleotides long) mediate epigenetic changes by integrating chromatin-modifying complexes, regulating post-transcriptional silencing, and genomic-imprinting [68,79]. piRNAs, a class of 21–35 nucleotides in length, are generated by a dicer-independent mechanism from long, single-stranded precursors. They protect germ cells by repressing active transposons [81].

Studies have shown that miRNAs play an imperative function in insulin signaling, glucose and lipid pathways, and the development of several metabolic disorders, including GDM [53,82,83]. Dysregulated placental miRNAs can be discharged into maternal circulation in GDM mothers [84]. A significant increase in lncRNA MEG8 before and during pregnancy was observed in GDM patients compared to healthy controls [85]. Additionally, the downregulation of plasma lncRNA SNHG17 has been implicated in GDM prevalence [86].

### 3.2. Epigenetics Bioactive Diets

Nutrition is an important environmental factor that influences gene expression through epigenetic programming, contributing to altering disease and health outcomes [9,12]. Moreover, the prenatal and early postnatal nutritional environment can significantly impact the offspring’s metabolic health [8]. Early exposure to bioactive dietary components can stimulate a defensive epigenetic mechanism throughout life. For example, it can induce long-term alteration in the DNA methylation profile that regulates health and disease susceptibility later in life. Nutrients can alter the expression of vital epigenetic regulators, including HAT, HDACs, DNMTs, and TET proteins, which in turn influence the presence of epigenetic substrates for catalytic reactions [12]. Epigenetics diets are a special group of diets that contain bioactive components to mediate gene expression and cellular function through epigenetic modifications, such as DNA methylation, histone modification, and non-coding RNAs. The components in epigenetic diets include, but are not limited to, catechin in green tea, resveratrol in berries and grape species, genistein in soy, allyl mercaptan in garlic, quercetin in citruses, and other phytochemicals or vitamins that can modify cellular epigenetic states [13,27].

One carbon metabolism utilizes nutrients, such as vitamins and amino acids, to fuel the metabolic pathway. Folate and methionine cycles, which are the two main constituents of the one carbon metabolism, recruit methyl groups to metabolites [87]. Folate, which is a water-soluble B vitamin, is essential for the synthesis of SAM (S-Adenosyl methionine), which regulates DNA and histone methylation through DNMTs and HMTs, respectively [9]. Folate and choline are well known methyl-donor nutrients which have been reported to regulate gene expressions during epigenomic establishment and maintenance and are essential for fetal growth and development. Thus, folate and choline supplements are antenatally recommended [12]. Deficiency in folic acids or choline can lead to global hypomethylation of the epigenome but can be reversed with the intake of methyl-donor nutrients [12]. Additionally, choline, a beneficial supplement required for organ and fetal development, is crosslinked with folate and methionine metabolism. Choline is delivered through the placenta to the fetus in utero. Hence, the placenta holds a large amount of choline as acetylcholine, which is essential for fetal growth and development [88]. Deficiency of intrauterine choline supply could lead to preterm birth and liver and brain damage [88,89].

Dietary polyphenols are present in most fruits and vegetables and classified based on their chemical structures. Dietary polyphenols include flavonoids (e.g., epigallocatechin-3-gallate [EGCG] in green tea), stilbenes (e.g., resveratrol in grapes and red wine), curcuminoids (e.g., curcumin in turmeric), and phenolic acids (e.g., protocatechuic acid in almonds). Flavonoids are further subdivided into isoflavones (e.g., daidzein and genistein in soy), anthocyanins (e.g., cyanidin-3-glucoside in blackberries), flavonols (e.g., quercetin in oranges), flavanols (e.g., EGCG in tea), and flavanones (e.g., hesperidin in oranges) [90]. These polyphenols can exert chemo-preventive roles against cancer and metabolic disorders by regulating histone or DNA modification patterns [27,90].

Tea is a common beverage that contains multiple polyphenols. Catechins are polyphenols predominantly found in green tea and include epicatechin (EC), epicatechin-3-gallate (ECG), epigallocatechin (EGC), and EGCG. Among them, EGCG is the most abundant bioactive component (>50%) in tea and has been well-studied for its anti-cancer effects by altering epigenetic landmarks leading to apoptosis, senescence, angiogenesis inhibition, and oxidative stress reduction in cancer cells. Mechanically, EGCG has been reported to act as DNMTs and HDACs inhibitors leading to demethylation, the reactivation of methylated-silenced genes, and the reversal of the global methylation state in cancer cells [91]. Similarly, EGCG can activate insulin receptor substrate and increase glucose uptake through the upregulation of GLUT4 and the regulation of miRNAs [90]. Catechins in green tea can induce weight loss by activating the AMPK pathway and fat oxidation [92]. Decaffeinate polyphenols from black tea, oolong tea, and green tea have been found to induce anti-inflammatory effects and weight loss and enhance adiponectin levels [93].

Genistein (GE) is a major bioactive isoflavones derived from soybean products, such as tofu, soy milk, and soy protein. Epidemiological data show a positive association between consumption of soybean products and low breast cancer prevalence among Asian women [94]. GE has been found to actively mediate histone acetylation, DNA methylation, and miRNAs, contributing to its anti-cancer properties [27]. It can repress hTERT, DNMT1, DNMT3a, and DNMT3b expressions in breast cancer cells, while enhancing the expression of p16, which is an essential tumor suppressor gene in glucose sensitive cancer cells [95,96]. Moreover, GE modifies histone markers by increasing the enrichment of H3K9me3 and decreasing H3K4me2 in the hTERT promoter region. Thus, GE can act as a potent demethylation agent to generate a hypomethylated state in the genome [95]. A recent study showed that a GE diet reduced the expression of tumor affiliated genes, such as NF-κB and Bcl-xL, and inhibited DNMTs, TETs, and HDACs in triple negative breast cancer (TNBC) [94]. Studies have shown the therapeutic effects of GE on ERα(-) breast cancer through its phytoestrogenic properties [97]. In addition to its anti-tumorigenic effects, GE has anti-inflammatory and antioxidant properties against metabolic disorders by mediating insulin sensitivity, fatty acid metabolism, and reactive oxygen species (ROS) [98,99]. GE can ameliorate glucose tolerance and insulin and glucagon ratios in type 2 diabetic models [100]. Daidzein is another natural isoflavone derived from soybean that has been shown to improve insulin sensitivity in obese mice via the upregulation of PPARγ and adiponectin alongside the inhabitation of TNF-α and adiposity [101].

Sulforaphane (SFN) is a secondary metabolite of isothiocyanates mostly found in cruciferous vegetables, such as cabbage, kale, broccoli, cauliflower, and radish [27]. Glucoraphanin is an inactive precursor of SFN that belongs to a class of glucosinolates. SFN is derived from the catabolic reaction of glucoraphanin mediated by myrosinase and gut microbiota through the mercapturic acid pathway [102]. SFN can act as an effective HDAC and DNMTs inhibitor leading to local and systemic histone acetylation and demethylation of regulatory genes [27]. Additionally, SFN has been found to indirectly restore miRNAs transcription by hypomethylating and enriching H3K4me1 at miRNA promoters [103]. SFN has antagonistic effects on obesity and type 2 diabetes. In mice, SFN can suppress high-fat diet-induced glucose intolerance and adipogenesis by upregulating browning-related gene expression in white adipose tissues and ROS reduction [104]. Thus, SFN has been identified as an effective therapeutic phytochemical in various cancers and metabolic diseases due to its potent anti-tumor and anti-inflammatory characteristics [105,106]. Prenatal and maternal consumption of SFN from broccoli sprouts also creates a potential chemo-preventive environment for fetal development [15].

Resveratrol is a natural polyphenol in grapes, berries, and peanuts, and it is abundant in grape skins and seeds. It can act as an effective DNMT and HDAC inhibitor by reversing methylation and acetylation states of regulatory genes [27]. It possesses anti-tumor, antioxidant, and anti-inflammatory properties that can target cancer and inflammatory diseases [107]. The protective effects of resveratrol include the suppression of oxidative stress markers in diabetic rats [108]. Resveratrol can also activate apoptotic signaling, alter gene expression leading to tumor reduction, and block the modulation of inflammatory activities in cancer cells [109]. Resveratrol can inhibit epithelial to mesenchymal transition (EMT) and invasion. At the molecular level, it can induce p53-mediated apoptosis and anti-proliferative activity and downregulate carcinogenic cellular signaling [110].

Dietary intervention via epigenetic diets can be used as alternative therapeutic strategies for cancer and metabolic diseases. The development and application of nutraceuticals from bioactive epigenetic dietary components is of the utmost importance for improving human health [27,111]. Intriguingly, the combination of polyphenols, isoflavones, and other phytochemicals from epigenetics diets have shown better preventive and therapeutic effects on human diseases and more genome-wide epigenetic alterations compared to a single bioactive dietary component, which may also point to a novel avenue for future disease prevention or therapy through a combinational dietary approach [106].

### 3.3. Effects of Maternal Epigenetics Diets in GDM

**Figure 2:**
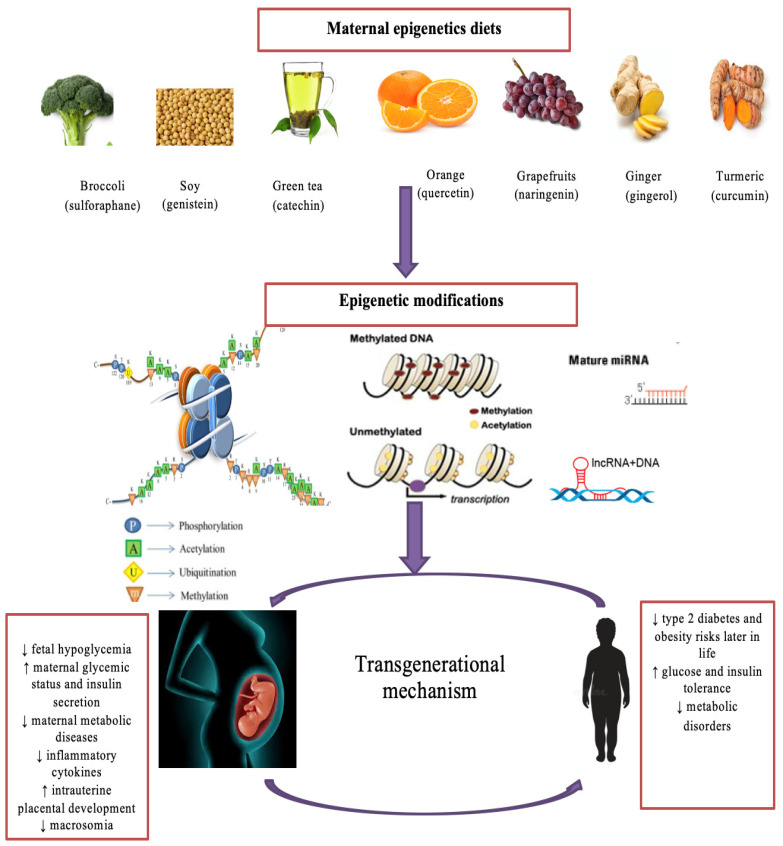
The impact of bioactive epigenetic dietary compounds on GDM. Maternal consumption of bioactive dietary components from a variety of epigenetics diets, such as sulforaphane in broccoli, genistein in soy, and catechins in green tea, can mediate epigenetic changes, such as histone modifications, DNA methylation patterns, and non-coding RNAs. Consequently, these epigenetic changes prevent metabolic disorders in GDM mothers, their developing fetuses, and their offspring later in life. Lastly, chromatin changes that influence metabolic disease susceptibility can be transferred from one generation to the next generation through epigenetic inherence from maternal epigenetics diets. miRNA refers to microRNA and lncRNA refers to long non-coding RNA. ↑ refers increase and ↓ refers decrease.

Maternal diet serves as an exclusive supply of nutrients required for placental progression and fetal development. Thus, imbalanced maternal nutrition could have a significant impact on offspring health [9,112]. Appropriate nutrition control through maternal diet has been utilized to manage impaired glycemic indexes in GDM [112]. Extensive studies have reported the protective effects of maternal epigenetics diets on fetal and maternal complications associated with GDM. For instance, methyl-donor nutrients, such as folate in oranges and choline in egg yolks, have been shown to improve insulin sensitivity and prevent fetal overgrowth, respectively. Controversially, there is an association between high-folate intake and GDM risk [113,114,115]. Catechin in tea activates anti-inflammatory cytokines, alleviates insulin resistance and hyperglycemia, and prevents fetal hypoglycemia and low birth weight [116,117]. In addition, the consumption of capsaicin found in chili may reduce cholesterol and triglycerides (TG) levels and prevent glucose and insulin dysfunction and fetal macrosomia in GDM mothers. [118]. Soy GE intake has been found to reduce fasting plasma glucose, insulin resistance, TG, very low-density lipoprotein, (VLDL) and fetal hyperbilirubinemia and improve reduced glutathione in GDM [119]. Gingerol in ginger and curcumin in turmeric play similar roles in suppressing fasting blood sugar, fasting insulin, and the insulin resistance index [120,121]. Maternal intake of curcumin restores glycogen levels and AMPK activation and contributes to reduced phosphorylated HDAC4 and glucose-6-phosphatase, leading to decreased birth weight [120]. Resveratrol may significantly reduce fasting glucose, cholesterol, TG, LDL, leptin, resistin, pro-inflammatory cytokines, and maternal body weight, but it may increase high-density lipoprotein HDL and adiponectin while improving insulin secretion [122]. Maternal supplementation of quercetin in tangerines reduces the thickness of placental labyrinth interhaemal membrane and upregulates placental adiponectin in GDM mothers [123]. Diallyl disulfide in garlic reduces fasting blood sugar and diastolic blood pressure [124]. Naringenin in grapefruits reduces maternal body weight and blood glucose, improves glucose and insulin tolerance, and significantly inhibits pro-inflammatory cytokines in GDM women [9]. These studies explicitly elucidate the beneficial effects of bioactive components from epigenetic diets on GDM complications and fetal development (Figure 2).

## 4. Gut Microbiome, Metabolome and Diet

The human body contains trillions of microflorae densely populated in the gastrointestinal (GI) tract. These gut microbiotas maintain a symbiotic or parasitic relationship with the human host and themselves [125]. Facultative and obligate anaerobic bacterial strains form the majority of the gut microbiome, such as phyla *Firmicutes*, *Bacteroidetes*, *Proteobacteria*, *Verrucomicrobia,* and *Actinobacteria* [126]. With its abundance and diversity, gut microbiota dynamically participates in host metabolism through facilitating digestive processes and mediating subsequent cellular processes [125]. The gut microbiota composition reflects healthy versus disease statuses. Dysbiosis is a disrupted gut microbiota composition that has been frequently associated with various chronic human diseases, including cancer, diabetes, and obesity [125]. Thus, gut microbial imbalance transferred from GDM mothers to infants could impact offspring’s metabolic outcomes later in life [36].

Importantly, gut microbiota influences health outcomes by synthesizing nutrients into metabolites that enter the host circulation [127]. Thus, gut dysbiosis can result in detrimental changes in metabolite profiles, resulting in metabolic disorders. For example, the oral administration of pathogenic bacteria, *Porphyromonas gingivalis*, disrupts gut microbiota composition and increases metabolites linked to diabetes and obesity [128]. Furthermore, nutrition and diets influence the composition and structure of gut microbiota [18]. The interaction of nutritional factors, gut microbiome, and metabolome play a critical role in GDM pathogenesis.

### 4.1. Establishment of Fetal Gut Microbiome

Although a few studies support the sterile womb theory, others have proven that the human gut microbiome usually establishes before birth and depends on maternal conditions during gestation. Maternal diets, perinatal body weight, health status, and other environmental factors collectively influence the gut microbial profiles of mothers and developing fetuses [18]. It appears that the maternal GI tract’s microbiota may be transferred to the developing fetus through the uteroplacental unit. Maternal intestinal microbial strains can colonize in fetal tissues, such as meconium, umbilical cords, placentas, and amniotic fluids [9,18]. An alteration in placental development in utero may lead to fetal gut dysbiosis. These findings reveal the impact of maternal gut microflora on fetal growth and development.

In addition, mother-breastmilk-infant linkage is important for establishing an early postnatal microbiome profile that defines both short- and long-term health outcomes for the offspring. It is widely accepted that human breast milk contains unique beneficial microbes and bioactive nutrients which exert protective effects against pathogenic strains present in infants’ intestinal epithelium. During lactation and pregnancy, maternal gut microbes translocate through cellular pathways to the mammary gland [22,129]. Newborns exposed to breastfeeding and milk formula show an increased prevalence of beneficial *Bifidobacterium* [129,130]. Thus, breast milk is a source of beneficial microbes and nutrients, which enhances infant health, growth, and development.

Notably, maternal nutritional status, antibiotic use, gestational age, and neonatal delivery mode have significant impacts on breastmilk and neonatal gut microbiome colonization [22,23,24]. Breastmilk microbiota from women with full term gestation expresses higher levels of beneficial *Bifidobacterium* species compared to those of mothers with preterm gestation. Similarly, *Bifidobacterium* species are increased in neonates from vaginal deliveries than those from caesarian sections [23,126]. The use of antibiotics during lactation and gestation alters bacterial richness and evenness (alpha diversity). Studies have shown that pups from antibiotics treated dams have a lower abundance of *Bacteroidetes acidifaciens*, *Bacteroides ovatus*, *Ruminococcus gnavus,* and *Parabacteroides distasonis*, but *Proteobacteria* was predominant in their gut [24]. Thus, maternal factors are primary determinants for infant gut microbial profiles, and bacterial communities in breast milk influence infant early gut microbial colonization and overall growth and development later in life [24,129].

### 4.2. Gut Microbiota-Produced Metabolites through Maternal Diets

Dietary choline is metabolized to betaine, which donates methyl groups to homocysteine to generate methionine. Betaine is converted to dimethylglycine (DMG), which is further synthesized to glycine. Choline is transported through the placental unit to the developing fetus. [131]. Gut microbes, such as *Firmicutes*, *Actinobacteria,* and *Proteobacteria* synthesize trimethylamines from dietary methylamines commonly found in red meat, egg yolk, and full-fat dairy products, which contain nutritional compounds, such as choline, L-carnitine, and phosphatidylcholine. The liver enzyme known as the flavin-containing monooxygenase further oxidizes trimethylamines into trimethylamine N-oxide (TMAO) [19]. An increased plasma TMAO concentration in early and mid-trimesters has been reported to be a risk factor for GDM [25]. Women with decreased GDM risk express a high betaine/choline ratio or a low DMG/betaine ratio during the second trimester [132].

Intestinal anaerobic bacteria metabolize dietary fibers into short chain fatty acids (SCFAs), such as butyric, propionic, valeric, caproic, and acetic acids. SCFAs act as signaling molecules of uteroplacental G-protein coupled receptors known as free-fatty acid receptors GPR41 (FFAR3) and GPR43 (FFAR2) and serve as a link between maternal diets, microbiome, and fetal and neonatal health [18,20]. For example, propionic acids regulate the metabolic programming of the developing fetus. Branched SCFAs, including isovaleric and isobutyric acids, are fermented by *Bacteroides* and *Clostridium* from branched amino acids, such as valine, leucine, and isoleucine. Dietary substrates and gut microflora determine the amount and synthesis of SCFAs in the GI tract. Moreover, SCFAs modulate brown tissue adiposity, fat storage, insulin resistance, and satiety hormones (ghrelin and leptin) [18,20]. The consumption of dietary fibers increases SCFAs levels, leading to enhanced insulin sensitivity and reduced dyslipidemia risk [20,21]. Newborns from GDM mothers show reduced SCFA-produced bacterial species, including *Lactobacillus*, *Flavonifractor*, *Erysipelotrichaceae*, and *Grammaproteobacteria,* and higher acetate levels [133]. Dysbiosis in pregnancy alters SCFAs levels [20,21]. Thus, SCFAs are implicated in the prevalence of metabolic diseases, such as GDM, obesity, and type 2 diabetes [20].

Intestinal gut microbiota also metabolizes bioactive nutrients from epigenetics diets. Gut microbiota participates in enzymatic processes during nutrient digestion. For example, microbially derived metabolites, such as β-thioglucosidases and β-glucosidases, convert glucoraphanin in broccoli sprouts and isoflavones in soybean into biologically active metabolites, such as SFN and GE, respectively. Intestinal reductase from certain microbes converts soy isoflavone daidzein to equol, which is a beneficial metabolite known for its estrogenic and antioxidant capacities [134]. Bifidobacterium synthesizes folate, and *Slackia equolifaciens* and *Adlercreutzia equolifaciens* synthesize resveratrol to produce glucuronides and sulfates [9,135,136]. Thus, microbial strains and microbiota-derived metabolites from epigenetic bioactive diets can be vertically conveyed from mothers to fetus and neonate, affecting offspring’s health.

### 4.3. The Roles of Gut Microbiome and Metabolome in Development of GDM

Gestational diabetes and other metabolic diseases are associated with a distorted gut microbiome and metabolomics profile. As stated earlier, gut microbiota is involved in host metabolism and physiological function, such as glucose, lipid, and insulin signaling that are highly associated with GDM etiology [137]. Gut microbiome synthesizes dietary substrates endogenously to generate metabolites, which are small, soluble compounds absorbed by the intestinal lumen [126]. Metabolites, such as SCFAs, bile acids, TMAO, tryptophan, indole derivatives, and branched amino acids, have been implicated in the pathology of metabolic disorders, including GDM [138]. A growing body of research has demonstrated the causal relationship between gut dysbiosis and GDM with an imbalance between commensal symbionts and pathobionts. GDM can cause gut dysbiosis which can be transferred to the developing fetus and newborn [126,137,138,139,140]. Dysbiosis among GDM women is clearly observed during the third trimester and continues until at least 8 months of conception [141]. Enrichment of *Ruminococcaceae*, *Parabacteroides distasonis*, *Prevotella*, *Desulfovibrio*, *Megamonas,* and *Phascolarctobacterium* was noted in GDM mothers compared to the controls [137,139]. The relative abundance of *P. distasonis*, *Klebsiella variicola*, *Catenibacterium mitsuokai*, *Coprococcus comes*, *Citrobacter* spp., *Methanobrevibacter smithii*, *Alistipes* spp., *Bifidobacterium* spp., and *Eubacterium* spp. was observed in women without GDM [140]. Insulin resistance was associated with an increased Firmicutes/Bacteroidetes ratio and reduced butyrate-producing bacteria, such as *Roseburia* and *Faecalibacterium prausnitzii* [19].

Furthermore, microbiome-derived metabolites have been implicated in GDM etiology. Increased isobutyric acid, isovaleric acid, valeric acid, caproic acid, and bile acid levels were positively associated with increased fasting glucose, TG, total cholesterol (TC), LDL, and reduced HDL, which are common metabolic profiles observed in GDM [139]. In addition to a distorted gut microbiota observed in GDM subjects, the upregulation of valine, allantoic acid, D-galactose, 3-methoxytyrosine, and D-glucose in fecal and urine samples were observed in GDM women compared to healthy controls [142]. Similarly, elevated levels of alanine, glutamic acid, and allantoin are associated with GDM. Disruption of glucose, amino acid, bile acids, and lipid metabolomic signatures are also found in GDM patients [142,143,144]. Thus, gut microbiome and metabolomic profiles may be potentially used as biomarkers for the early detection of GDM pathogenesis and progression [126,138].

Maternal GDM may impact fetal and neonatal microbiome and metabolome. Meconium from newborns with GDM mothers showed increased *Proteobacteria* and *Actinobacteria* phyla and reduced *Prevotella* and *Lactobacillus*. As opposed to GDM neonates, newborns from healthy mothers showed increased beneficial *Bacteroidetes* and *Butyrivibrio*, which are butyrate producers [145]. Additionally, elevated Firmicutes and depleted Proteobacteria at the phylum level and elevated *Streptococcaceae*, *Clostridium,* and *Rothia* at the genus level were observed in GDM newborns [146]. A metabolomic analysis from GDM neonates showed similar serum metabolites imbalance found in GDM mothers [146]. These studies suggest that prenatal gut microbial and metabolite aberrations in GDM women can influence offspring gut microbiome and metabolomic profiles through the fetoplacental system and maternal–neonatal transmission. Importantly, this pinpoints a positive correlation between GDM dysbiosis and the onset and development of fetal and neonatal metabolic diseases.

## 5. Interplay between Maternal Diets, Gut Microbiome, Metabolome, and Epigenome on GDM Pathogenesis

Studies have reported the roles of microbial-synthesized metabolites derived from diets on gene expression profiles, DNA methylation, histone modifications, and miRNAs [9,20,147]. For instance, SCFAs, which are beneficial metabolites derived from microbial fermentation of indigestible foods, such as dietary fiber, may regulate epigenetic processes and influence lipogenesis, gluconeogenesis, and inflammation [147]. Gut microbiota produce biological active metabolites, such as folate and acetyl CoA, which can regulate epigenetic markers [9]. Growing evidence indicates that maternal nutritional status can influence fetal and neonatal epigenetic regulation through the establishment of gut microbiome and metabolomics profiles [9,148]. Thus, the dynamic interaction between maternal diets, gut microbiota, metabolome, and host epigenome may determine individual disease susceptibility later in life.

### 5.1. Maternal Diets Alter Gut Microbiome and Metabolome

Nutrition status during pregnancy is a significant modulator for maternal and neonatal health [19]. Maternal diets consumed prenatally and postnatally greatly affect offspring’s intestinal microbiome and metabolism. Intestinal microbes may initiate inauspicious metabolic regulation via maternofetal interface in the presence of unhealthy maternal diets which result in a disrupted glucose and lipid metabolism, leading to an increased risk of neonatal metabolic abnormalities [20,24].

The development of GDM has been well known to relate to the consumption of unhealthy diets, such as high fat, high sugar, and low dietary fiber diets. GDM is positively linked to other metabolic disorders, such as obesity, overweight, and type 2 diabetes, due to the similar pathways involved in glucose, lipid, and insulin metabolism [18,20]. Thus, maternal unhealthy diets may adversely alter the composition and diversity of intestinal microbes and the abundance of microbial metabolites. Additionally, altered gut microbiome and metabolomics profiles can result in maternal chronic inflammatory responses, such as increased pro-inflammatory cytokines transmitted through the placenta to the fetus, thereby distorting fetal and neonatal health, growth, and development [20,149]. It is well known that the use of probiotics during pregnancy can reduce the risk of GDM and other metabolic diseases [9,18,21]. Probiotics contain live beneficial bacteria, such as *Bifidobacterium*, which can use oligosaccharides as their main carbon source [18,21]. Prebiotics utilize indigestible diets to enhance the activity and growth of beneficial intestinal microbes. The combination of probiotics and prebiotics is termed synbiotics [21,150]. Notably, prebiotics supplementation during pregnancy reduces GWG, fasting glucose and insulin resistance in GDM patients. Maternal prebiotics intake also reduces neonatal fasting plasma glucose, body fat, and leptin levels [151]. Particularly, probiotics can modulate maternal and fetal gut microbiota colonization [18,21]. Thus, prenatal probiotics and prebiotics can promote overall maternal and fetal and neonatal metabolic outcomes [9,18,21,151].

Studies have reported the impact of bioactive compounds from epigenetic diets on metabolic disorders. Fecal microbiota transplanted from allicin-treated mice into obese mice inhibit excessive body weight and fat mass, improve glucose and lipid homeostasis, and increase SCFAs-producing bacterial strains [152]. Combinatorial effects of dietary bioactive nutrients affect gut microbial and metabolites composition [153]. These findings suggest that bioactive components from diets can alter gut microbiome and metabolomics profiles. Table 1 summarized how diets or nutrition factors affect the gut microbiome and metabolite profiles.

### 5.2. Crosstalk between Maternal Diets, Gut Microbiome, Metabolome and Epigenome in the Pathogenesis of Metabolic Disorders

Recent studies have shown the impact of gut microbiome and metabolites on epigenetic changes which contribute to the pathogenesis of metabolic disorders. The epigenetic alternations are more prominent at the early infant stages due to microbial colonization influenced by breastfeeding, delivery mode, and antibiotics use [147]. Biologically active metabolites, such as folate, choline, SCFAs, TMAO, and biotin are involved in epigenetic reprogramming by altering transcriptional machinery during early development [18,147,148].

Biotin is a soluble vitamin that is mainly synthesized by Bacteroides in the gut. Biotin is also involved in chromatin remodeling by modifying histones as a substrate for biotinyl transfer [174,175]. At the transcriptional level, biotin regulates glucokinase and phosphoenolpyruvate carboxykinase, which are the key enzymes in glycolysis and gluconeogenesis, indicating that biotin regulates glucose signaling and lipid metabolism [28,175]. Biotin deficiency is associated with obesity and type 2 diabetes. An impaired bacterial production of biotin is mostly observed in metabolic diseases [28].

Furthermore, SCFAs have been widely reported as HDAC inhibitors [20,147]. In mice, consumption of high fat diet results in lower levels of SCFAs, which negatively alters histone acetylation and abolishes microbiota-dependent H4 acetylation. Acetate, one of the SCFAs, can be directly converted to acetyl CoA, which is an acetyl-donor for HATs [29]. Butyrate is an important SCFA produced by beneficial bacterial strains, such as Roseburia spp., Eubacterium, and Bifidobacterium, and it is considered an important HDAC inhibitor. Both acetate and butyrate can increase histone acetylation and reverse silenced genes [20,29]. Butyrate can initiate anti-inflammation by repressing NF-κB and INF-γ and upregulating PPARγ [18,176]. Butyrate regulates DNA methylation by inducing DNMT1 downregulation, demethylating some tumor suppressor genes such as p21 and p16 [148], and regulating miRNA expression [177].

**Figure 3:**
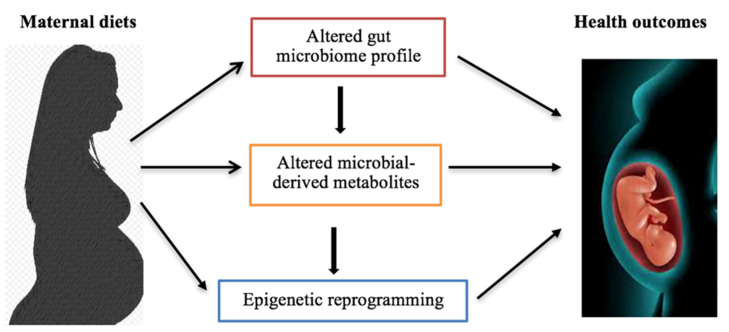
Maternal diets influence microbiome-metabolomics-epigenetics interplay, resulting in health outcome changes. Maternal diets can alter the gut microbial profile and microbiota-synthesized metabolites. Additionally, the transplacental transfer of maternal intestinal microbes and metabolites to the developing fetus can influence epigenetic reprogramming progresses in fetuses. Principally, this crosstalk influences offspring health outcomes, such as metabolic disease susceptibility, later in life.

Metabolites, such as folate, betaine, vitamin B12, and choline, are involved in 5-methyltetrahydrofolate metabolism which provides one-carbon unit for the conversion of homocysteine to methionine. The latter is utilized for SAM generation, which is the methyl donor for DNA and histone methylation [148,178]. Studies have shown relationships between choline, microbiota, epigenetic regulation, and metabolic disorders [178,179]. Choline deficient can alter global DNA methylation which promotes diet-induced metabolic disease susceptibility [178]. TMAO synthesized from choline can impact epigenetic changes through the increased production of ROS, causing deamination or depurination of bases, especially during fetal development. Consequently, this triggers DNA repair mechanisms and the loss of methylated cytosine (epimutation) [148,180]. Excess ROS induces epigenetic remodification that intersects SAM and the antioxidant pathway in response to oxidative stress [180]. Thus, prenatal, perinatal, and early postnatal diets may influence early-life epigenetic reprogramming processes through, at least in part, gut microbiome-metabolomics-epigenetics interface, consequently influencing fetal and neonatal health, growth, and later life development (Figure 3).

## 6. Recent Clinical Trials through Maternal Diet in the Prevention of GDM

Dietary intervention to prevent GDM during pregnancy is becoming a medical advancement. A number of human studies have been reported regarding the use of maternal dietary supplements to reduce the risk of GDM (Table 2). The therapeutic effects of probiotics and/or Mediterranean diets on metabolic diseases have been well established [18,181,182]. In a recent study, co-supplementation of selenium (200µg/day) and probiotics (2 × 10^9^ CFU/day each of *Lactobacillus acidophilus*, *Bifidobacterium bifidum*, *B. lactis*, and *B. longum*) for 6 weeks significantly impacted maternal glycemic status by reducing fasting glucose and insulin resistance. Similarly, these supplements decreased TG, TC, and LDL levels and increased PPAR-γ expression [181]. In addition, prenatal consumption of Mediterranean diets has been found to reduce maternal–fetal complications and reduce GDM risk [183].

Furthermore, epigenetics diets exhibit a compelling impact on GDM. Soy diets containing 35% animal protein, 35% soy protein, and 30% other plant protein consumed by GDM women for 6 weeks significantly reduced fasting plasma glucose, insulin resistance, and TG and increased insulin sensitivity and antioxidant effects in GDM women. Notably, maternal soy consumption suppressed neonatal hyperbilirubinemia [119]. Daily intake of 500 mg of green tea EGCG deceased plasma fasting glucose and insulin resistance in GDM mothers and inhibited neonatal hypoglycemia and macrosomia in newborns [117]. A combination of dietary polyphenols in blueberry and soluble fiber intake during pregnancy reduced GWG and improved glycemic index in GDM women [185]. These data demonstrate the clinical implication of maternal bioactive diets in GDM management and prevention.

As aforementioned, prenatal and postnatal diets significantly influence fetal and neonatal growth and development. Thus, a healthy dietary pattern is recommended for pregnant mothers. According to the 2020–2025 USDA dietary guidelines for Americans, pregnant and lactating mothers require a daily intake of 2.5–3.5 cups of vegetables and 1.5–2.5 cups of fruits. However, exact consumption depends on the calorie intake required to maintain a healthy BMI during pregnancy. Further, the nutrition guideline recommends a sufficient dietary intake of folate, iron, choline, iodine, seafoods, and beverages before and during pregnancy [189]. This indicates that a beneficial dietary plan is urgently needed to help prevent nutrient deficiencies and diet-induced metabolic diseases like GDM, resulting in a healthy pregnancy outcome for both mother and baby. Notably, these guidelines should be reinforced through more clinical trials and translational research.

## 7. Conclusions

GDM is a global obstetrical complication positively associated with other metabolic syndromes which influence maternal and fetal health. It has been well established that epigenetics diets during pregnancy and lactation can influence maternal gut microbiome and metabolomics profiles, which can be vertically transmitted to the developing fetus in utero. The microbiota–metabolite crosstalk modulates fetal and neonatal epigenetic reprogramming and has a significant impact on offspring’s metabolic health later in life. Thus, maternal dietary intervention can potentially reduce the risk of developing GDM and its associated complications in mothers and infants by minimizing gut dysbiosis. Clinical implementation of maternal bioactive diets can be utilized in GDM management and prevention. Future studies on unexplored bioactive compounds in epigenetics diets, combinatorial approaches, and time of exposure will be important to explore more efficacious strategies for the prevention of GDM in the affected women and their babies.

## Figures and Tables

**Table 1:** Effects of bioactive dietary components on gut microbial and metabolite profiles.

Diets	Microbial Profiles	Metabolite Profiles
High fat diet	↓ *Bacteroides, Prevotella, Lactobacillus* [19,154], and *Bifidobacterium* [19]	↓ SCFAs (acetic, propionic acids, butyric, and isobutyric) [154,155]
	↑ *Clostridium* [151] and *Firmicutes/Bacteroidetes* ratio [154,155]	↑ 3-hydroxybutyrate, acetone and acetoacetate, and 2-oxoisocaproate [151]
		↑ isoleucine, leucine, and valine [151] and ↓ alanine, proline [151]
		↑ o-phosphocholine, cytidine [151]
Curcumin, e.g., turmeric	↑ *Akkermansia, Bacteroides, Parabacteroides, Alistipes,* and *Alloprevotella* [155]	↑ acetate, propionic acid, butyric, and isobutyric [155]
	↓ *Firmicutes/Bacteroidetes* ratio*, Desulfovibrio,* and *Ruminococcaceae* [155]	
Sulforaphane, e.g., kale	↑ *Bacteroides fragilis, Clostridium cluster 1* [156], *Bacteroidetes*, and *Lactobacillus* [153]	↑ butyric acid [156]
	↓ *Actinobacteria, Proteobacteria,* and *Lactococcus* [153]	
Green tea polyphenol	↑ *Bifidobacterium* [157] and *Bacteroidetes* [153]	↑ acetate and propionate [157]
	↓ *Clostridium perfringens, C. difficile*, [157,158], and *Proteobacteria* [153]	↑ isobutyrate, valerate, and hexanoate [153]
Prebiotics, e.g., banana	↑ *Bifidobacterium* and *Bacteroides/Prevotella* spp. [151]	↑ myo-inositol [151]
	↓ *Methanobrevibacter* spp., *Roseburia* spp., *Clostridium coccoides*, and *C. leptum* [151]	↑ arginine, ornithine, citrulline, and proline [151]
Diallyl disulfide, e.g., garlic	↓ *Parabacteroides, Faecalibacterium, Escherichia-Shigella,* and *Streptococcus* [159]	↑ 4-(2-aminophenyl)-2,4-dioxobutanoic acid, and kynurenic acid [159]
	↑ *Ruminiclostridium, Oscillibacter, Ruminococcaceae,* and *Prevotellaceae* [159]	↑ 5-hydroxyindoleacetic acid, quinoline-4,8-diol, 4-(2-amino-3-hydroxyphenyl)-2,4-dioxobutanoic acid, 3-methyldioxyindole, 2-formaminobenzoylacetate, lipoxin A4, and cholic acids [159]
	↑ *Bifidobacterium, Bacteroidetes*, and *Lactobacillus* [152]	
	↑ *Bacteroidetes/Firmicutes* ratio [152]	
		↑ SCAFs (acetate, valine) [152]
Soy	↑ *Bacteroidetes, proteobacteria, Enterococcus, Lactobacilli,* and *Bifidobacterium* [160,161,162]	↑ SCAFs (butyric acid and lactic acid) [160,161,163]
	↓ *Clostridia, Enterobacteria, Firmicutes* [160,163]	
	↑ *Blautia* and *Veillonellaceae* [164]	
Resveratrol, e.g., grapes	↑ *Lactobacillus, Bacteroidetes/Firmicutes* ratio, and *Bifidobacterium* [135,165]	3, 4′-dihydroxy-trans-stilbene and 3,4′-dihydroxy benzyl (lunularin) [135]
	↓ *Enterococcus faecalis* [135], *Clostridia spp.*, *Parabacteroides distasonis,* and *Gracilibacter thermotolerans* [165]	glucuronide, sulfate [136,166]
		↓ TMAO [167]
Quercetin, e.g., citrus fruits	↓ *Firmicutes/Bacteroidetes* ratio, *Eubacterium cylindroides* [165], *Escherichia-shigella* [168], *Desulfovibrio,* and *Helicobacter* [169]	↑ butyrate [168,169], acetate, and propionate [169]
Pectin, e.g., apples	↑ *Lactobacillus, Bifidobacterium* [170,171,172] and *Bacteroidetes* [171]	↑ acetate [171,173], propionate, and butyrate [171,172,173]
	↓ *C. perfringens, Enterobacteriaceae, Pseudomonas* [170], and *Firmicutes* [171]	

↑ refers increase and ↓ refers decrease.

**Table 2:** Clinical studies in maternal diets on GDM outcomes.

Clinical Trial	Dose/Supplement	Health Outcome on GDM	Reference
Effect of the Mediterranean diet supplemented with extra virgin oil and pistachios on GDM	Daily consumption of ≥40 mL of extra virgin oil and 25–30 g of pistachios in addition to basic Med Diet recommendations for 16 weeks	↓ maternal fasting blood glucose, insulin resistance, and GWG ↓ neonatal LGA	[184]
Dietary blueberry and fiber supplements for GDM women	280 g whole blueberries and 12 g soluble fiber daily for 18 weeks	↓ maternal weight gain and blood glucose	[185]
Effect of DASH diet on GDM	Daily intake of a diet rich in fruits, vegetables, whole grains, low-fat dairy products, and a diet low in saturated fats, cholesterol, and refined grains and sweets for 4 weeks	Improved pregnancy and fetal health outcomes Reduced the need for insulin therapy ↓ fetal macrosomia	[186]
Probiotic supplements	Daily intake of probiotic capsules (2 × 10^9^ CFU/g each of ^a^ *Lactobacillus* spp. and ^b^ *Bifidobacterium* spp.)	↑ PPAR-γ, HDL and antioxidant capacity ↓ TNF-α, fasting glucose, insulin resistance, TG, and VLDL	[187]
Myo-inositol supplements	4 g/day throughout pregnancy	Reduced the risk of preterm birth, macrosomia, and maternal weight gain	[188]

DASH: dietary approaches to stop hypertension. CFU: colony-forming unit. ^a^ *Lactobacillus acidophilus*, *L. casei*, and *L. fermentum.* ^b^ *Bifidobacterium bifidum*. ↑ refers increase and ↓ refers decrease.

## Data Availability

Not applicable.

## References

[1] Plows J.F., Stanley J.L., Baker P.N., Reynolds C.M., Vickers M.H. (2018). The Pathophysiology of Gestational Diabetes Mellitus. Int. J. Mol. Sci..

[2] Wang H., Li N., Chivese T., Werfalli M., Sun H., Yuen L., Hoegfeldt C.A., Powe C.E., Immanuel J., Karuranga S. (2022). IDF Diabetes Atlas: Estimation of Global and Regional Gestational Diabetes Mellitus Prevalence for 2021 by International Association of Diabetes in Pregnancy Study Group’s Criteria. Diabetes Res. Clin. Pr..

[3] Yamada H., Hirayama Kato E., Tsuruga R., Ebina Y., Kobashi G., Sagawa T., Makita Z., Koike T., Fujimoto S. (2001). Insulin Response Patterns Contribute to Different Perinatal Risks in Gestational Diabetes. Gynecol. Obstet. Investig..

[4] Caughey A.B., Turrentine M. (2018). ACOG Practice Bulletin No. 190 Summary: Gestational Diabetes Mellitus. Obstet. Gynecol..

[5] Alejandro E., Mamerto T., Chung G., Villavieja A., Gaus N., Morgan E., Pineda-Cortel M. (2020). Gestational Diabetes Mellitus: A Harbinger of the Vicious Cycle of Diabetes. Int. J. Mol. Sci..

[6] Aplin J.D., Myers J.E., Timms K., Westwood M. (2020). Tracking placental development in health and disease. Nat. Rev. Endocrinol..

[7] Gómez-Roig M.D., Pascal R., Cahuana M.J., García-Algar O., Sebastiani G., Andreu-Fernández V., Martínez L., Rodríguez G., Iglesia I., Ortiz-Arrabal O. (2021). Environmental Exposure during Preg-nancy: Influence on Prenatal Development and Early Life: A Comprehensive Review. Fetal Diagn Ther..

[8] Chu A.H.Y., Godfrey K.M. (2020). Gestational Diabetes Mellitus and Developmental Programming. Ann. Nutr. Metab..

[9] Li Y. (2018). Epigenetic Mechanisms Link Maternal Diets and Gut Microbiome to Obesity in the Offspring. Front. Genet..

[10] Kapur K., Kapur A., Hod M. (2020). Nutrition Management of Gestational Diabetes Mellitus. Ann. Nutr. Metab..

[11] Li Y., Saldanha S.N., Tollefsbol T.O. (2014). Impact of Epigenetic Dietary Compounds on Transgenerational Prevention of Human Diseases. AAPS J..

[12] Tiffon C. (2018). The Impact of Nutrition and Environmental Epigenetics on Human Health and Disease. Int. J. Mol. Sci..

[13] Li S., Chen M., Li Y., Tollefsbol T.O. (2019). Prenatal epigenetics diets play protective roles against environmental pollution. Clin. Epigenetics.

[14] Chen M., Li S., Arora I., Yi N., Sharma M., Li Z., Tollefsbol T.O., Li Y. (2022). Maternal soybean diet on prevention of obesity-related breast cancer through early-life gut microbiome and epigenetic regulation. J. Nutr. Biochem..

[15] Li Y., Buckhaults P., Li S., Tollefsbol T. (2018). Temporal Efficacy of a Sulforaphane-Based Broccoli Sprout Diet in Prevention of Breast Cancer through Modulation of Epigenetic Mechanisms. Cancer Prev. Res..

[16] Chen M., Li S., Srinivasasainagendra V., Sharma M., Li Z., Tiwari H., Tollefsbol T.O., Li Y. (2022). Maternal soybean genistein on pre-vention of later-life breast cancer through inherited epigenetic regulations. Carcinogenesis..

[17] Zhu W., Shen Y., Liu J., Fei X., Zhang Z., Li M., Chen X., Xu J., Zhu Q., Zhou W. (2020). Epigenetic alternations of microRNAs and DNA methylation contribute to gestational diabetes mellitus. J. Cell. Mol. Med..

[18] Miko E., Csaszar A., Bodis J., Kovacs K. (2022). The Maternal-Fetal Gut Microbiota Axis: Physiological Changes, Dietary Influence, and Modulation Possibilities. Life.

[19] Ponzo V., Fedele D., Goitre I., Leone F., Lezo A., Monzeglio C., Finocchiaro C., Ghigo E., Bo S. (2019). Diet-Gut Microbiota Interactions and Gestational Diabetes Mellitus (GDM). Nutrients.

[20] Ziętek M., Celewicz Z., Szczuko M. (2021). Short-chain fatty acids, maternal microbiota, and metabolism in pregnancy. Nutrients.

[21] Li X., Yu D., Wang Y., Yuan H., Ning X., Rui B., Lei Z., Yuan J., Yan J., Li M. (2021). The Intestinal Dysbiosis of Mothers with Gestational Diabetes Mellitus (GDM) and Its Impact on the Gut Microbiota of Their Newborns. Can. J. Infect. Dis. Med. Microbiol..

[22] Verduci E., Giannì M., Vizzari G., Vizzuso S., Cerasani J., Mosca F., Zuccotti G. (2021). The Triad Mother-Breast Milk-Infant as Predictor of Future Health: A Narrative Review. Nutrients.

[23] Khodayarpardo P., Pascual L.M., Collado M.C., Martinez-Costa C. (2014). Impact of lactation stage, gestational age and mode of delivery on breast milk microbiota. J. Perinatol..

[24] Nyangahu D.D., Lennard K.S., Brown B., Darby M., Wendoh J.M., Havyarimana E., Smith P., Butcher J., Stintzi A., Mulder N.J. (2018). Disruption of maternal gut microbiota during gestation alters offspring microbiota and immunity. Microbiome.

[25] Li P., Zhong C., Li S., Sun T., Huang H., Chen X., Zhu Y., Hu X., Peng X., Zhang X. (2018). Plasma concentration of trimethylamine-N-oxide and risk of gestational diabetes mellitus. Am. J. Clin. Nutr..

[26] Latino C., Gianatti E.J., Mehta S., Lo J., Devine A., Christophersen C. (2022). Does a high dietary intake of resistant starch affect glycaemic control and alter the gut microbiome in women with gestational diabetes? A randomised control trial protocol. BMC Pregnancy Childbirth.

[27] Hardy T.M., Tollefsbol T.O. (2011). Epigenetic diet: Impact on the epigenome and cancer. Epigenomics.

[28] Belda E., Voland L., Tremaroli V., Falony G., Adriouch S., Assmann K.E., Prifti E., Aron-Wisnewsky J., Debédat J., Le Roy T. (2022). Impairment of gut microbial biotin metabolism and host biotin status in severe obesity: Effect of biotin and prebiotic sup-plementation on improved metabolism. Gut.

[29] Krautkramer K.A., Kreznar J.H., Romano K.A., Vivas E.I., Barrett-Wilt G.A., Rabaglia M.E., Keller M.P., Attie A.D., Rey F.E., Denu J.M. (2016). Diet-Microbiota Interactions Mediate Global Epigenetic Programming in Multiple Host Tissues. Mol. Cell.

[30] Brown J., Alwan N.A., West J., Brown S., McKinlay C.J., Farrar D., Crowther C.A. (2017). Lifestyle interventions for the treatment of women with gestational diabetes. Cochrane Database Syst. Rev..

[31] Mustad V.A., Huynh D.T., López-Pedrosa J.M., Campoy C., Rueda R. (2020). The Role of Dietary Carbohydrates in Gestational Diabetes. Nutrients.

[32] Nguyen-Ngo C., Jayabalan N., Salomon C., Lappas M. (2019). Molecular pathways disrupted by gestational diabetes mellitus. J. Mol. Endocrinol..

[33] Djelmiš J., Desoye G., Ivaniševic M. (2005). Diabetology of Pregnancy. Frontiers in Diabetes.

[34] Franzago M., Fraticelli F., Stuppia L., Vitacolonna E. (2019). Nutrigenetics, epigenetics and gestational diabetes: Consequences in mother and child. Epigenetics.

[35] Catalano P.M., Ehrenberg H.M. (2006). The short and long term implications of maternal obesity on the mother and her off-spring. British J. Obstet. Gynecology..

[36] Choudhury A.A., Devi Rajeswari V. (2021). Gestational diabetes mellitus—A metabolic and reproductive disorder. Biomed Pharmacother..

[37] Catalano P.M., Nizielski S.E., Shao J., Preston L., Qiao L., Friedman J.E. (2002). Downregulated IRS-1 and PPARγ in obese women with gestational diabetes: Relationship to free fatty acids during pregnancy. Am. J. Physiol. Endocr. Metab..

[38] Stettler C., Christ E., Diem P. (2016). Novelties in Diabetes. Endocr. Dev..

[39] Ehrenberg H.M., Mercer B.M., Catalano P.M. (2004). The influence of obesity and diabetes on the prevalence of macrosomia. Am. J. Obstet. Gynecol..

[40] Kc K., Shakya S., Zhang H. (2015). Gestational Diabetes Mellitus and Macrosomia: A Literature Review. Ann. Nutr. Metab..

[41] Wendland E.M., Torloni M.R., Falavigna M., Trujillo J., Dode M.A., Campos M.A., Duncan B.B., Schmidt M.I. (2012). Gestational diabetes and pregnancy outcomes–a systematic review of the World Health Organization (WHO) and the International Association of Diabetes in Pregnancy Study Groups (IADPSG) di-agnostic criteria. BMC Pregnancy Childbirth.

[42] Su W.-J., Chen Y.-L., Huang P.-Y., Shi X.-L., Yan F.-F., Chen Z., Yan B., Song H.-Q., Lin M.-Z., Li X.-J. (2019). Effects of Prepregnancy Body Mass Index, Weight Gain, and Gestational Diabetes Mellitus on Pregnancy Outcomes: A Population-Based Study in Xiamen, China, 2011. Ann. Nutr. Metab..

[43] Nguyen M.T., Ouzounian J.G. (2021). Evaluation and Management of Fetal Macrosomia. Obstet. Gynecol. Clin. North Am..

[44] El Hajj N., Schneider E., Lehnen H., Haaf T. (2014). Epigenetics and life-long consequences of an adverse nutritional and diabetic in-trauterine environment. Reproduction.

[45] Sweeting A.N., Wong J., Appelblom H., Ross G.P., Kouru H., Williams P.F., Sairanen M., Hyett J.A. (2019). A Novel Early Pregnancy Risk Prediction Model for Gestational Diabetes Mellitus. Fetal Diagn. Ther..

[46] Pan X.-F., Huang Y., Li X., Wang Y., Ye Y., Chen H., Marklund M., Wen Y., Liu Y., Zeng H. (2021). Circulating fatty acids and risk of gestational diabetes mellitus: Prospective analyses in China. Eur. J. Endocrinol..

[47] Pippen J., Stetson B., Doherty L., Varner M.W., Casey B.M., Reddy U.M., Wapner R.J., Rouse D.J., Tita A.T.N., Thorp J.M. (2022). Eunice Kennedy Shriver National Institute of child health human development maternal-fetal medicine units network. Neonatal birthweight, infant feeding, and childhood metabolic markers. Am. J. Perinatol..

[48] Mustaniemi S., Vääräsmäki M., Eriksson J.G., Gissler M., Laivuori H., Ijäs H., Bloigu A., Kajantie E., Morin-Papunen L. (2018). Polycystic ovary syndrome and risk factors for gestational diabetes. Endocr. Connect..

[49] Ruchat S.-M., Hivert M.-F., Bouchard L. (2013). Epigenetic programming of obesity and diabetes by in utero exposure to gestational diabetes mellitus. Nutr. Rev..

[50] Silva-Zolezzi I., Samuel T.M., Spieldenner J. (2017). Maternal nutrition: Opportunities in the prevention of gestational diabetes. Nutr. Rev..

[51] Sweet C.B., Grayson S., Polak M. (2013). Management Strategies for Neonatal Hypoglycemia. J. Pediatr. Pharmacol. Ther..

[52] Brumbaugh D.E., Tearse P., Cree-Green M., Fenton L.Z., Brown M., Scherzinger A., Reynolds R., Alston M., Hoffman C., Pan Z. (2013). Intrahepatic Fat Is Increased in the Neonatal Offspring of Obese Women with Gestational Diabetes. J. Pediatr..

[53] Lorenzo-Almorós A., Hang T., Peiró C., Soriano-Guillén L., Egido J., Tuñón J., Lorenzo Ó. (2019). Predictive and diagnostic biomarkers for gestational diabetes and its associated metabolic and cardiovascular diseases. Cardiovasc. Diabetol..

[54] Agarwal P., Morriseau T.S., Kereliuk S.M., Doucette C.A., Wicklow B.A., Dolinsky V.W. (2018). Maternal obesity, diabetes during preg-nancy and epigenetic mechanisms that influence the developmental origins of cardiometabolic disease in the offspring. Crit. Rev. Clin. Lab. Sci..

[55] Law P.-P., Holland M.L. (2019). DNA methylation at the crossroads of gene and environment interactions. Essays Biochem..

[56] Sun C., Fan J.-G., Qiao L. (2015). Potential Epigenetic Mechanism in Non-Alcoholic Fatty Liver Disease. Int. J. Mol. Sci..

[57] Deshpande S.S., Balasinor N.H. (2018). Placental Defects: An Epigenetic Perspective. Reprod. Sci..

[58] Nelissen E.C., van Montfoort A.P., Dumoulin J.C., Evers J.L. (2010). Epigenetics and the placenta. Hum. Reprod. Updat..

[59] Caniçais C., Vasconcelos S., Ramalho C., Marques C.J., Dória S. (2021). Deregulation of imprinted genes expression and epigenetic regulators in placental tissue from intrauterine growth restriction. J. Assist. Reprod. Genet..

[60] Meng H., Cao Y., Qin J., Song X., Zhang Q., Shi Y., Cao L. (2015). DNA Methylation, Its Mediators and Genome Integrity. Int. J. Biol. Sci..

[61] Moore L.D., Le T., Fan G. (2013). DNA methylation and its basic function. Neuropsychopharmacology.

[62] Li S., Tollefsbol T.O. (2021). DNA methylation methods: Global DNA methylation and methylomic analyses. Methods.

[63] Howe C.G., Cox B., Fore R., Jungius J., Kvist T., Lent S., Miles H.E., Salas L.A., Rifas-Shiman S., Starling A.P. (2020). Maternal Gestational Diabetes Mellitus and Newborn DNA Methylation: Findings from the Pregnancy and Childhood Epigenetics Consortium. Diabetes Care.

[64] Nomura Y., Lambertini L., Rialdi A., Lee M., Ba E.Y.M., Grabie M., Bs I.M., Huynh N., Finik J., Davey M. (2014). Global Methylation in the Placenta and Umbilical Cord Blood from Pregnancies With Maternal Gestational Diabetes, Preeclampsia, and Obesity. Reprod. Sci..

[65] Ruchat S.-M., Houde A.-A., Voisin G., St-Pierre J., Perron P., Baillargeon J.-P., Gaudet D., Hivert M.-F., Brisson D., Bouchard L. (2013). Gestational diabetes mellitus epigenetically affects genes predominantly involved in metabolic diseases. Epigenetics.

[66] Wang L., Fan H., Zhou L., Wu Y., Lu H., Luo J. (2018). Altered expression of PGC-1 α and PDX1 and their methylation status are associated with fetal glucose metabolism in gestational diabetes mellitus. Biochem. Biophys. Res. Commun..

[67] Barlow D.P., Bartolomei M.S. (2014). Genomic imprinting in mammals. Cold Spring Harb Perspect Biol..

[68] Inbar-Feigenberg M., Choufani S., Butcher D.T., Roifman M., Weksberg R. (2013). Basic concepts of epigenetics. Fertil. Steril..

[69] Petry C., Mooslehner K., Prentice P., Hayes M., Nodzenski M., Scholtens D., Hughes I., Acerini C., Ong K., Lowe W. (2017). Associations between a fetal imprinted gene allele score and late pregnancy maternal glucose concentrations. Diabetes Metab..

[70] Zhang Y., Sun Z., Jia J., Du T., Zhang N., Tang Y., Fang Y., Fang D. (2021). Overview of Histone Modification. Adv. Exp. Med. Biol..

[71] Zhou B.-R., Bai Y. (2019). Chromatin structures condensed by linker histones. Essays Biochem..

[72] Tolsma T.O., Hansen J.C. (2019). Post-translational modifications and chromatin dynamics. Essays Biochem..

[73] Ramazi S., Allahverdi A., Zahiri J. (2020). Evaluation of post-translational modifications in histone proteins: A review on histone modification defects in developmental and neurological disorders. J. Biosci..

[74] Li Y., Seto E. (2016). HDACs and HDAC Inhibitors in Cancer Development and Therapy. Cold Spring Harb. Perspect. Med..

[75] Schuettengruber B., Bourbon H.-M., Di Croce L., Cavalli G. (2017). Genome Regulation by Polycomb and Trithorax: 70 Years and Counting. Cell.

[76] Hepp P., Hutter S., Knabl J., Hofmann S., Kuhn C., Mahner S., Jeschke U. (2018). Histone H3 Lysine 9 Acetylation is Downregulated in GDM Placentas and Calcitriol Supplementation Enhanced This Effect. Int. J. Mol. Sci..

[77] Li Y., Li G., Guo X., Yao H., Wang G., Li C. (2020). Non-coding RNA in bladder cancer. Cancer Lett..

[78] Panni S., Lovering R.C., Porras P., Orchard S. (2020). Non-coding RNA regulatory networks. Biochim. Biophys. Acta Gene Regul. Mech..

[79] Li Y. (2021). Modern epigenetics methods in biological research. Methods.

[80] Moen G.H., Sommer C., Prasad R.B., Sletner L., Groop L., Qvigstad E., Birkeland K.I. (2017). Mechanisms in Endocrinology: Ep-igenetic modifications and gestational diabetes: A systematic review of published literature. Eur. J. Endocrinol..

[81] Ozata D.M., Gainetdinov I., Zoch A., O’Carroll D., Zamore P.D. (2019). PIWI-interacting RNAs: Small RNAs with big functions. Nat. Rev. Genet..

[82] Sliwinska A., Kasinska M.A., Drzewoski J. (2017). MicroRNAs and metabolic disorders - where are we heading?. Arch. Med. Sci..

[83] Liu Z.-N., Jiang Y., Liu X.-Q., Yang M.-M., Chen C., Zhao B.-H., Huang H.-F., Luo Q. (2021). MiRNAs in Gestational Diabetes Mellitus: Potential Mechanisms and Clinical Applications. J. Diabetes Res..

[84] Dias S., Pheiffer C., Abrahams Y., Rheeder P., Adam S. (2018). Molecular Biomarkers for Gestational Diabetes Mellitus. Int. J. Mol. Sci..

[85] Zhang W., Cao D., Wang Y., Ren W. (2021). LncRNA MEG8 is upregulated in gestational diabetes mellitus (GDM) and predicted kidney injury. J. Diabetes its Complicat..

[86] Li J., Du B., Geng X., Zhou L. (2021). lncRNA SNHG17 is Downregulated in Gestational Diabetes Mellitus (GDM) and Has Predictive Values. Diabetes Metab. Syndr. Obes. Targets Ther..

[87] Mentch S.J., Locasale J.W. (2016). One-carbon metabolism and epigenetics: Understanding the specificity. Ann. N. Y. Acad. Sci..

[88] Zeisel S.H. (2006). Choline: Critical Role During Fetal Development and Dietary Requirements in Adults. Annu. Rev. Nutr..

[89] Bernhard W., Poets C.F., Franz A.R. (2019). Choline and choline-related nutrients in regular and preterm infant growth. Eur. J. Nutr..

[90] Santangelo C., Zicari A., Mandosi E., Scazzocchio B., Mari E., Morano S., Masella R. (2016). Could gestational diabetes mellitus be managed through dietary bioactive compounds? Current knowledge and future perspectives. Br. J. Nutr..

[91] Khan M.A., Hussain A., Sundaram M.K., Alalami U., Gunasekera D., Ramesh L., Hamza A., Quraishi U. (2015). (−)-Epigallocatechin-3-gallate reverses the expression of various tumor-suppressor genes by inhibiting DNA methyltransferases and histone deacetylases in human cervical cancer cells. Oncol. Rep..

[92] Rothenberg D.O., Zhou C., Zhang L. (2018). A Review on the Weight-Loss Effects of Oxidized Tea Polyphenols. Molecules.

[93] Heber D., Zhang Y., Yang J., Ma J.E., Henning S.M., Li Z. (2014). Green Tea, Black Tea, and Oolong Tea Polyphenols Reduce Visceral Fat and Inflammation in Mice Fed High-Fat, High-Sucrose Obesogenic Diets. J. Nutr..

[94] Sharma M., Arora I., Chen M., Wu H., Crowley M.R., Tollefsbol T.O., Li Y. (2021). Therapeutic Effects of Dietary Soybean Genistein on Triple-Negative Breast Cancer via Regulation of Epigenetic Mechanisms. Nutrients.

[95] Li Y., Liu L., Andrews L.G., Tollefsbol T.O. (2009). Genistein depletes telomerase activity through cross-talk between genetic and epi-genetic mechanisms. Int. J. Cancer.

[96] Li Y., Liu L., Tollefsbol T.O. (2010). Glucose restriction can extend normal cell lifespan and impair precancerous cell growth through epigenetic control of *hTERT* and *p16* expression. FASEB J..

[97] Li Y., Meeran S.M., Patel S.N., Chen H., Hardy T.M., Tollefsbol T.O. (2013). Epigenetic reactivation of estrogen receptor-α (ERα) by genistein enhances hormonal therapy sensitivity in ERα-negative breast cancer. Mol. Cancer.

[98] Choi J.S., Koh I.-U., Song J. (2012). Genistein reduced insulin resistance index through modulating lipid metabolism in ovariectomized rats. Nutr. Res..

[99] Behloul N., Wu G. (2013). Genistein: A promising therapeutic agent for obesity and diabetes treatment. Eur. J. Pharmacol..

[100] Gilbert E.R., Liu D. (2013). Anti-diabetic functions of soy isoflavone genistein: Mechanisms underlying its effects on pancreatic β-cell function. Food Funct..

[101] Sakamoto Y., Naka A., Ohara N., Kondo K., Iida K. (2014). Daidzein regulates proinflammatory adipokines thereby improving obe-sity-related inflammation through PPARγ. Mol. Nutr. Food Res..

[102] Vanduchova A., Anzenbacher P., Anzenbacherova E. (2019). Isothiocyanate from Broccoli, Sulforaphane, and Its Properties. J. Med. Food..

[103] Gao L., Cheng D., Yang J., Wu R., Li W., Kong A.-N. (2018). Sulforaphane epigenetically demethylates the CpG sites of the miR-9-3 promoter and reactivates miR-9-3 expression in human lung cancer A549 cells. J. Nutr. Biochem..

[104] Liu Y., Fu X., Chen Z., Luo T., Zhu C., Ji Y., Bian Z. (2021). The Protective Effects of Sulforaphane on High-Fat Diet-Induced Obesity in Mice Through Browning of White Fat. Front. Pharmacol..

[105] Li Z., Li Y. (2021). Bioactive Dietary Compounds and Epigenetics in Women’s Reproductive Cancers. Compr. Pharmacol..

[106] Sharma M., Tollefsbol T.O. (2022). Combinatorial epigenetic mechanisms of sulforaphane, genistein and sodium butyrate in breast cancer inhibition. Exp. Cell Res..

[107] Galiniak S., Aebisher D., Bartusik-Aebisher D. (2019). Health benefits of resveratrol administration. Acta Biochim. Pol..

[108] Sedlak L., Wojnar W., Zych M., Wyględowska-Promieńska D., Mrukwa-Kominek E., Kaczmarczyk-Sedlak I. (2018). Effect of Resveratrol, a Dietary-Derived Polyphenol, on the Oxidative Stress and Polyol Pathway in the Lens of Rats with Streptozotocin-Induced Diabetes. Nutrients.

[109] Udenigwe C., Ramprasath V.R., Aluko R.E., Jones P.J.H. (2008). Potential of resveratrol in anticancer and anti-inflammatory therapy. Nutr. Rev..

[110] Kim C.W., Hwang K.A., Choi K.C. (2016). Anti-metastatic potential of resveratrol and its metabolites by the inhibition of epitheli-al-mesenchymal transition, migration, and invasion of malignant cancer cells. Phytomedicine.

[111] Divella R., Daniele A., Savino E., Paradiso A. (2020). Anticancer Effects of Nutraceuticals in the Mediterranean Diet: An Epigenetic Diet Model. Cancer Genom. Proteom..

[112] Shimron-Nachmias L., Frishman S., Hod M. (2006). Dietary management of diabetic pregnancy. Harefuah.

[113] Chen X., Zhang Y., Chen H., Jiang Y., Wang Y., Wang D., Li M., Dou Y., Sun X., Huang G. (2021). Association of Maternal Folate and Vitamin B12 in Early Pregnancy with Gestational Diabetes Mellitus: A Prospective Cohort Study. Diabetes Care.

[114] Setola E., Monti L., Galluccio E., Palloshi A., Fragasso G., Paroni R., Magni F., Sandoli E., Lucotti P., Costa S. (2004). Insulin resistance and endothelial function are improved after folate and vitamin B12 therapy in patients with metabolic syndrome: Relationship between homocysteine levels and hyperinsulinemia. Eur. J. Endocrinol..

[115] Nam J., Greenwald E., Jack-Roberts C., Ajeeb T.T., Malysheva O.V., Caudill M.A., Axen K., Saxena A., Semernina E., Nanobashvili K. (2017). Choline prevents fetal overgrowth and normalizes placental fatty acid and glucose metabolism in a mouse model of maternal obesity. J. Nutr. Biochem..

[116] Esmaeelpanah E., Razavi B.M., Hosseinzadeh H. (2021). Green tea and metabolic syndrome: A 10-year research update review. Iran J. Basic Med. Sci..

[117] Zhang H., Su S., Yu X., Li Y. (2017). Dietary epigallocatechin 3-gallate supplement improves maternal and neonatal treatment outcome of gestational diabetes mellitus: A double-blind randomised controlled trial. J. Hum. Nutr. Diet..

[118] Yuan L.-J., Qin Y., Wang L., Zeng Y., Chang H., Wang J., Wang B., Wan J., Chen S.-H., Zhang Q.-Y. (2016). Capsaicin-containing chili improved postprandial hyperglycemia, hyperinsulinemia, and fasting lipid disorders in women with gestational diabetes mellitus and lowered the incidence of large-for-gestational-age newborns. Clin. Nutr..

[119] Jamilian M., Asemi Z. (2015). The Effect of Soy Intake on Metabolic Profiles of Women with Gestational Diabetes Mellitus. J. Clin. Endocrinol. Metab..

[120] Lu X., Wu F., Jiang M., Sun X., Tian G. (2019). Curcumin ameliorates gestational diabetes in mice partly through activating AMPK. Pharm. Biol..

[121] Hajimoosayi F., Jahanian Sadatmahalleh S., Kazemnejad A., Pirjani R. (2020). Effect of ginger on the blood glucose level of women with gestational diabetes mellitus (GDM) with impaired glucose tolerance test (GTT): A randomized double-blind place-bo-controlled trial. BMC Complement Med. Ther..

[122] Zhang G., Wang X., Ren B., Zhao Q., Zhang F. (2021). The Effect of Resveratrol on Blood Glucose and Blood Lipids in Rats with Gesta-tional Diabetes Mellitus. Evid. Based Complement Alternat. Med..

[123] Mahabady M.K., Shamsi M.M., Ranjbar R., Tabandeh M.R., Khazaeel K. (2021). Quercetin improved histological structure and upregulated adiponectin and adiponectin receptors in the placenta of rats with gestational diabetes mellitus. Placenta.

[124] Faroughi F., Charandabi S.M., Javadzadeh Y., Mirghafourvand M. (2018). Effects of Garlic Pill on Blood Glucose Level in Borderline Gestational Diabetes Mellitus: A Triple Blind, Randomized Clinical Trial. Iran. Red Crescent Med. J..

[125] Milani C., Duranti S., Bottacini F., Casey E., Turroni F., Mahony J., Belzer C., Delgado Palacio S., Arboleya Montes S., Mancabelli L. (2017). The First Microbial Colonizers of the Human Gut: Composition, Activities, and Health Implications of the Infant Gut Microbiota. Microbiol. Mol. Biol. Rev..

[126] Lamichhane S., Sen P., Dickens A.M., Orešič M., Bertram H.C. (2018). Gut metabolome meets microbiome: A methodological perspective to understand the relationship between host and microbe. Methods.

[127] Wilmanski T., Rappaport N., Earls J.C., Magis A.T., Manor O., Lovejoy J., Omenn G.S., Hood L., Gibbons S.M., Price N.D. (2019). Blood metabolome predicts gut microbiome α-diversity in humans. Nat. Biotechnol..

[128] Kato T., Yamazaki K., Nakajima M., Date Y., Kikuchi J., Hase K., Ohno H., Yamazaki K. (2018). Oral Administration of Porphyromonas gingivalis Alters the Gut Microbiome and Serum Metabolome. Msphere.

[129] Lyons K.E., Ryan C.A., Dempsey E.M., Ross R.P., Stanton C. (2020). Breast Milk, a Source of Beneficial Microbes and Associated Benefits for Infant Health. Nutrients.

[130] Bittinger K., Zhao C., Li Y., Ford E., Friedman E.S., Ni J., Kulkarni C.V., Cai J., Tian Y., Liu Q. (2020). Bacterial colonization reprograms the neonatal gut metabolome. Nat. Microbiol..

[131] Friesen R.W., Novak E.M., Hasman D., Innis S.M. (2007). Relationship of Dimethylglycine, Choline, and Betaine with Oxoproline in Plasma of Pregnant Women and Their Newborn Infants. J. Nutr..

[132] Gong X., Du Y., Li X., Yang J., Zhang X., Wei Y., Zhao Y. (2021). Maternal Plasma Betaine in Middle Pregnancy Was Associated with Decreased Risk of GDM in Twin Pregnancy: A Cohort Study. Diabetes Metab. Syndr. Obesity Targets Ther..

[133] Soderborg T.K., Carpenter C.M., Janssen R.C., Weir T.L., Robertson C.E., Ir D., Young B.E., Krebs N.F., Hernandez T.L., Barbour L.A. (2020). Gestational Diabetes Is Uniquely Associated with Altered Early Seeding of the Infant Gut Microbiota. Front. Endocrinol..

[134] Mayo B., Vázquez L., Flórez A.B. (2019). Equol: A Bacterial Metabolite from The Daidzein Isoflavone and Its Presumed Beneficial Health Effects. Nutrients.

[135] Qiao Y., Sun J., Xia S., Tang X., Shi Y., Le G. (2014). Effects of resveratrol on gut microbiota and fat storage in a mouse model with high-fat-induced obesity. Food Funct..

[136] Chaplin A., Carpéné C., Mercader J. (2018). Resveratrol, Metabolic Syndrome, and Gut Microbiota. Nutrients.

[137] Hasain Z., Mokhtar N.M., Kamaruddin N.A., Ismail N.A.M., Razalli N.H., Gnanou J.V., Ali R.A.R. (2020). Gut Microbiota and Gestational Diabetes Mellitus: A Review of Host-Gut Microbiota Interactions and Their Therapeutic Potential. Front. Cell. Infect. Microbiol..

[138] Agus A., Clément K., Sokol H. (2021). Gut microbiota-derived metabolites as central regulators in metabolic disorders. Gut.

[139] Gao Y., Chen H., Li J., Ren S., Yang Z., Zhou Y., Xuan R. (2022). Alterations of gut microbiota-derived metabolites in gestational diabetes mellitus and clinical significance. J. Clin. Lab. Anal..

[140] Kuang Y.S., Lu J.H., Li S.H., Li J.H., Yuan M.Y., He J.R., Chen N.N., Xiao W.Q., Shen S.Y., Qiu L. (2017). Connections between the human gut microbiome and gestational diabetes mellitus. Gigascience.

[141] Crusell M.K., Hansen T.H., Nielsen T., Allin K.H., Rühlemann M.C., Damm P., Vestergaard H., Rørbye C., Jørgensen N.R., Christiansen O.B. (2018). Gestational diabetes is associated with change in the gut microbiota composition in third trimester of pregnancy and postpartum. Microbiome.

[142] Wang X., Liu H., Li Y., Huang S., Zhang L., Cao C., Baker P.N., Tong C., Zheng P., Qi H. (2020). Altered gut bacterial and metabolic signatures and their interaction in gestational diabetes mellitus. Gut Microbes.

[143] Bentley-Lewis R., Huynh J., Xiong G., Lee H., Wenger J., Clish C., Nathan D., Thadhani R., Gerszten R. (2015). Metabolomic profiling in the prediction of gestational diabetes mellitus. Diabetologia.

[144] Hou W., Meng X., Zhao A., Zhao W., Pan J., Tang J., Huang Y., Li H., Jia W., Liu F. (2018). Development of Multimarker Diagnostic Models from Metabolomics Analysis for Gestational Diabetes Mellitus (GDM). Mol. Cell. Proteom..

[145] Su M., Nie Y., Shao R., Duan S., Jiang Y., Wang M., Xing Z., Sun Q., Liu X., Xu W. (2018). Diversified gut microbiota in newborns of mothers with gestational diabetes mellitus. PLOS ONE.

[146] Chen T., Qin Y., Chen M., Zhang Y., Wang X., Dong T., Chen G., Sun X., Lu T., Xia Y. (2021). Gestational diabetes mellitus is associated with the neonatal gut microbiota and metabolome. BMC Med..

[147] Sierra A.C., Ramos-Lopez O., Riezu-Boj J.I., Milagro F.I., Martinez J.A. (2019). Diet, Gut Microbiota, and Obesity: Links with Host Genetics and Epigenetics and Potential Applications. Adv. Nutr..

[148] Sharma M., Li Y., Stoll M.L., Tollefsbol T.O. (2020). The Epigenetic Connection Between the Gut Microbiome in Obesity and Diabetes. Front. Genet..

[149] Myatt L., Maloyan A. (2016). Obesity and Placental Function. Semin. Reprod. Med..

[150] Gibson G.R., Roberfroid M.B. (1995). Dietary Modulation of the Human Colonic Microbiota: Introducing the Concept of Prebiotics. J. Nutr..

[151] Paul H.A., Bomhof M.R., Vogel H.J., Reimer R.A. (2016). Diet-induced changes in maternal gut microbiota and metabolomic profiles influence programming of offspring obesity risk in rats. Sci. Rep..

[152] Zhang C., He X., Sheng Y., Yang C., Xu J., Zheng S., Liu J., Xu W., Luo Y., Huang K. (2020). Allicin-induced host-gut microbe interactions improves energy homeostasis. FASEB J..

[153] Sharma M., Arora I., Stoll M.L., Li Y., Morrow C.D., Barnes S., Berryhill T.F., Li S., Tollefsbol T.O. (2020). Nutritional combinatorial impact on the gut microbiota and plasma short-chain fatty acids levels in the prevention of mammary cancer in Her2/neu estrogen receptor-negative transgenic mice. PLoS ONE.

[154] Hsu C.N., Hou C.Y., Lee C.T., Chan J.Y., Tain Y.L. (2019). The interplay between maternal and post-weaning high-fat diet and gut microbiota in the developmental programming of hypertension. Nutrients.

[155] Li S., You J., Wang Z., Liu Y., Wang B., Du M., Zou T. (2021). Curcumin alleviates high-fat diet-induced hepatic steatosis and obesity in association with modulation of gut microbiota in mice. Food Res. Int..

[156] He C., Huang L., Lei P., Liu X., Li B., Shan Y. (2018). Sulforaphane Normalizes Intestinal Flora and Enhances Gut Barrier in Mice with BBN-Induced Bladder Cancer. Mol. Nutr. Food Res..

[157] Okubo T., Ishihara N., Oura A., Serit M., Kim M., Yamamoto T., Mitsuoka T. (1992). In Vivo Effects of Tea Polyphenol Intake on Human Intestinal Microflora and Metabolism. Biosci. Biotechnol. Biochem..

[158] Lee H.C., Jenner A.M., Low C.S., Lee Y.K. (2006). Effect of tea phenolics and their aromatic fecal bacterial metabolites on intestinal mi-crobiota. Res. Microbiol..

[159] Hu W., Huang L., Zhou Z., Yin L., Tang J. (2022). Diallyl Disulfide (DADS) Ameliorates Intestinal Candida albicans Infection by Modu-lating the Gut microbiota and Metabolites and Providing Intestinal Protection in Mice. Front. Cell Infect. Microbiol..

[160] Huang H., Krishnan H.B., Pham Q., Yu L.L., Wang T.T.Y. (2016). Soy and Gut Microbiota: Interaction and Implication for Human Health. J. Agric. Food Chem..

[161] Tamura K., Sasaki H., Shiga K., Miyakawa H., Shibata S. (2019). The Timing Effects of Soy Protein Intake on Mice Gut Microbiota. Nutrients.

[162] Gill I.R., Uno J.K. (2016). The impact of dietary soy on Gut microbiome. FASEB.

[163] Zhu Y., Shi X., Lin X., Ye K., Xu X., Li C., Zhou G. (2017). Beef, Chicken, and Soy Proteins in Diets Induce Different Gut Microbiota and Metabolites in Rats. Front. Microbiol..

[164] Dioletis E., Paiva R.S., Kaffe E., Secor E.R., Weiss T.R., Fields M.R., Ouyang X., Ali A. (2021). The fermented soy beverage Q-CAN® plus induces beneficial changes in the oral and intestinal microbiome. BMC Nutr..

[165] Etxeberria U., Arias N., Boqué N., Macarulla M., Portillo M., Martínez J., Milagro F. (2015). Reshaping faecal gut microbiota composition by the intake of trans-resveratrol and quercetin in high-fat sucrose diet-fed rats. J. Nutr. Biochem..

[166] Zhang B., Xu Y., Lv H., Pang W., Wang J., Ma H., Wang S. (2021). Intestinal pharmacokinetics of resveratrol and regulatory effects of resveratrol metabolites on gut barrier and gut microbiota. Food Chem..

[167] Hsu C.N., Hou C.Y., Chang-Chien G.P., Lin S., Yang H.W., Tain Y.L. (2020). Perinatal resveratrol therapy prevents hypertension pro-grammed by maternal chronic kidney disease in adult male offspring: Implications of the gut microbiome and their me-tabolites. Biomedicines.

[168] Shi T., Bian X., Yao Z., Wang Y., Gao W., Guo C. (2020). Quercetin improves gut dysbiosis in antibiotic-treated mice. Food Funct..

[169] Porras D., Nistal E., Martínez-Flórez S., Pisonero-Vaquero S., Olcoz J.L., Jover R., González-Gallego J., García-Mediavilla M.V., Sánchez-Campos S. (2017). Protective effect of quercetin on high-fat diet-induced non-alcoholic fatty liver disease in mice is mediated by modulating intestinal microbiota imbalance and related gut-liver axis activation. Free. Radic. Biol. Med..

[170] Shinohara K., Ohashi Y., Kawasumi K., Terada A., Fujisawa T. (2010). Effect of apple intake on fecal microbiota and metabolites in humans. Anaerobe.

[171] Hu H., Zhang S., Liu F., Zhang P., Muhammad Z., Pan S. (2019). Role of the Gut Microbiota and Their Metabolites in Modulating the Cholesterol-Lowering Effects of Citrus Pectin Oligosaccharides in C57BL/6 Mice. J. Agric. Food Chem..

[172] Li W., Zhang K., Yang H. (2018). Pectin Alleviates High Fat (Lard) Diet-Induced Nonalcoholic Fatty Liver Disease in Mice: Possible Role of Short-Chain Fatty Acids and Gut Microbiota Regulated by Pectin. J. Agric. Food Chem..

[173] Marounek M., Volek Z., Synytsya A., Čopíková J. (2007). Effect of pectin and amidated pectin on cholesterol homeostasis and cecal metabolism in rats fed a high-cholesterol diet. Physiol. Res..

[174] Rodriguez-Melendez R., Zempleni J. (2003). Regulation of gene expression by biotin (review). J. Nutr. Biochem..

[175] Dakshinamurti K. (2005). Biotin--a regulator of gene expression. J. Nutr. Biochem..

[176] Berni Canani R., Di Costanzo M., Leone L. (2012). The epigenetic effects of butyrate: Potential therapeutic implications for clinical practice. Clin. Epigenetics.

[177] Bishop K.S., Xu H., Marlow G. (2017). Epigenetic Regulation of Gene Expression Induced by Butyrate in Colorectal Cancer: Involvement of MicroRNA. Genet. Epigenetics.

[178] Romano K.A., Martinez-Del Campo A., Kasahara K., Chittim C.L., Vivas E.I., Amador-Noguez D., Balskus E.P., Rey F.E. (2017). Metabolic, Epigenetic, and Transgenerational Effects of Gut Bacterial Choline Consumption. Cell Host Microbe.

[179] Romano K.A., Rey F.E. (2018). Is maternal microbial metabolism an early-life determinant of health?. Lab. Anim..

[180] Ortega Ávila J.G., Echeverri I., de Plata C.A., Castillo A. (2015). Impact of oxidative stress during pregnancy on fetal epigenetic patterns and early origin of vascular diseases. Nutr. Rev..

[181] Amirani E., Asemi Z., Taghizadeh M. (2022). The effects of selenium plus probiotics supplementation on glycemic status and serum lipoproteins in patients with gestational diabetes mellitus: A randomized, double-blind, placebo-controlled trial. Clin. Nutr. ESPEN.

[182] Karamali M., Dadkhah F., Sadrkhanlou M., Jamilian M., Ahmadi S., Tajabadi-Ebrahimi M., Jafari P., Asemi Z. (2016). Effects of probiotic supplementation on glycaemic control and lipid profiles in gestational diabetes: A randomized, double-blind, placebo-controlled trial. Diabetes Metab..

[183] Assaf-Balut C., de la Torre N.G., Fuentes M., Durán A., Bordiú E., del Valle L., Valerio J., Jiménez I., Herraiz M.A., Izquierdo N. (2018). A High Adherence to Six Food Targets of the Mediterranean Diet in the Late First Trimester is Associated with a Reduction in the Risk of Materno-Foetal Outcomes: The St. Carlos Gestational Diabetes Mellitus Prevention Study. Nutrients.

[184] Assaf-Balut C., Garcia De La Torre N., Durán A., Fuentes M., Bordiú E., Del Valle L., Familiar C., Ortolá A., Jiménez I., Herraiz M.A. (2017). A Mediterranean diet with additional extra virgin olive oil and pistachios reduces the incidence of gestational diabetes mellitus (GDM): A randomized controlled trial: The St. Carlos GDM prevention study. PLoS ONE.

[185] Basu A., Feng D., Planinic P., Ebersole J.L., Lyons T.J., Alexander J.M. (2021). Dietary Blueberry and Soluble Fiber Supplementation Reduces Risk of Gestational Diabetes in Women with Obesity in a Randomized Controlled Trial. J. Nutr..

[186] Asemi Z., Samimi M., Tabassi Z., Esmaillzadeh A. (2014). The effect of DASH diet on pregnancy outcomes in gestational diabetes: A randomized controlled clinical trial. Eur. J. Clin. Nutr..

[187] Babadi M., Khorshidi A., Aghadavood E., Samimi M., Kavossian E., Bahmani F., Mafi A., Shafabakhsh R., Satari M., Asemi Z. (2019). The Effects of Probiotic Supplementation on Genetic and Metabolic Profiles in Patients with Gestational Diabetes Mellitus: A Randomized, Double-Blind, Placebo-Controlled Trial. Probiotics Antimicrob. Proteins.

[188] Santamaria A., Alibrandi A., Di Benedetto A., Pintaudi B., Corrado F., Facchinetti F., D’Anna R. (2018). Clinical and metabolic outcomes in pregnant women at risk for gestational diabetes mellitus supplemented with myo-inositol: A secondary analysis from 3 RCTs. Am. J. Obstet. Gynecol..

[189] U.S. Department of Agriculture, U.S. Department of Health and Human Services (2020). Dietary Guidelines for Americans, 2020–2025, 9th Edition. DietaryGuidelines.gov.

